# Variability of the Ability of Complex Microbial Communities to Exclude Microbes Carrying Antibiotic Resistance Genes in Rabbits

**DOI:** 10.3389/fmicb.2019.01503

**Published:** 2019-07-02

**Authors:** Caroline Stéphanie Achard, Véronique Dupouy, Suzanne Siviglia, Nathalie Arpaillange, Laurent Cauquil, Alain Bousquet-Mélou, Olivier Zemb

**Affiliations:** ^1^GenPhySE, INRA, ENVT, Université de Toulouse, Toulouse, France; ^2^Lallemand SAS, Blagnac, France; ^3^InTheRes, INRA, ENVT, Université de Toulouse, Toulouse, France

**Keywords:** rabbit, gut microbiota, antibiotic resistance gene, 16S sequencing, competitive exclusion

## Abstract

Reducing antibiotic use is a necessary step toward less antibiotic resistance in livestock, but many antibiotic resistance genes can persist for years, even in an antibiotic-free environment. In this study, we investigated the potential of three fecal complex microbial communities from antibiotic-naive does to drive the microbiota of kits from antibiotic-exposed dams and outcompete bacteria-carrying antibiotic-resistant genes. The fecal complex microbial communities were either orally delivered or simply added as fresh fecal pellets in four to five nests that were kept clean from maternal feces. Additionally, four nests were cleaned for the maternal feces and five nests were handled according to the common farm practice (i.e., cleaning once a week) as controls. At weaning, we measured the relative abundance of 26 antibiotic resistance genes, the proportion of *Enterobacteriaceae* resistant to tetracycline and sulfonamide antibiotics, and the taxonomic composition of the microbiota by sequencing the 16S rRNA genes of one kit per nest. Changing the surrounding microbes of the kits can hinder the transmission of antibiotic resistance genes from one generation to the next, but the three communities widely differed in their ability to orient gut microbes and in their impact on antibiotic resistance genes. The most efficient delivery of the microbial community reduced the proportion of resistant *Enterobacteria* from 93 to 9%, decreased the relative abundance of eight antibiotic resistance genes, and changed the gut microbes of the kits at weaning. The least efficient did not reduce any ARG or modify the bacterial community. In addition, adding fecal pellets was more efficient than the oral inoculation of the anaerobic suspension derived from these fecal pellets. However, we were unable to predict the outcome of the exclusion from the data of the donor does (species composition and abundance of antibiotic resistance genes). In conclusion, we revealed major differences between microbial communities regarding their ability to exclude antibiotic resistance genes, but more work is needed to understand the components leading to the successful exclusion of antibiotic resistance genes from the gut. As a consequence, studies about the impact of competitive exclusion should use several microbial communities in order to draw general conclusions.

## Introduction

Microbes exposed to antibiotics in livestock may develop resistance mechanisms, including a variety of targets such as efflux systems, drug modifiers or changes in the target’s configuration (Davies and Davies, 2010). As a matter of fact, the abundance of antibiotic resistance genes (ARGs) in the gut of livestock varies between countries. For example, Chinese pigs have more ARGs than their French conspecifics (Xiao et al., 2016). Furthermore, the antibiotic resistance genes found in the human gut seem to coincide with the antibiotics authorized for livestock in Spain and Denmark (Forslund et al., 2013), thereby raising the question of ARGs potentially transferred from bacteria inhabiting the gut of livestock to bacteria inhabiting the human gut.

To make matters worse, livestock harboring antibiotic resistance genes in their gut could represent a constant potential threat to public health despite the constant decrease of antibiotic use. Indeed, some ARGs incur almost no fitness cost for the bacteria. For example, *Campylobacter* spontaneously evolved a resistance against macrolides and was not outcompeted by its sensitive kin in an antibiotic-free environment (Zeitouni et al., 2012). In human volunteers, some ARGs may persist for 2 years after the selection pressure via antibiotics, so that the commensal microbiota might be a major reservoir of ARGs (Jernberg et al., 2007, 2010).

Considering that dams do receive antibiotics, the ARG persistence in the gut microbiota of dams implies that the kits could inherit antibiotic-resistant microbes that persist until slaughter at 63 days, even if the kits are raised without antibiotics. It should be noted that dams are particularly exposed to antibiotics. For example, a French doe during a reproductive cycle receives on average 1.4 ± 0.7 antibiotic treatments per day, while a growing rabbit receives 0.8 ± 0.3 (Fortun-Lamothe et al., 2011) during the fattening period.

Luckily, the sensitivity of the kits’ microbiota to their surrounding microbes could also be the key to exclusion-based approaches. The idea of using a complex microbial community to remediate against a bacterial species of interest was first described in 1973 (Nurmi and Rantala, 1973) to outcompete *Salmonella* in broilers. Competitive exclusion was also successfully adapted to the exclusion of antibiotic-resistant *E. coli* in broilers (Hofacre et al., 2002; Nuotio et al., 2013) and in mice after an antibiotic treatment (Ubeda et al., 2013). Today, fecal transplantation using complex microbial communities is also used in hospitals to cure *Clostridium difficile* infections (Chapman et al., 2016) and lyophilization protocols are developed to facilitate its routine use (Staley et al., 2017). However, studies using complex microbial communities for competitive exclusion remain relatively scarce for mammalian livestock and it is difficult to draw general conclusions. For example, *Salmonella* could be outcompeted *in vitro* from communities taken from pigs (Anderson et al., 1999; Genovese et al., 2003) but microbial communities used for competitive exclusion have also been reported to sometimes increase the antibiotic resistance of *E. coli in vivo* (Kim et al., 2005).

In this study, we investigated the potential of three fecal complex microbial communities from antibiotic-naive does to drive the microbiota of kits from antibiotic-exposed dams and to outcompete bacteria carrying antibiotic resistant genes. The fecal complex microbial communities were either orally delivered or simply added as fresh fecal pellets in the nest. We measured the antibiotic resistance genes, the *Enterobacteriaceae* resistant to antibiotics, and the taxonomic composition of the microbiota of the kits at weaning.

## Materials and Methods

### Animals

The kits for which the competitive exclusion was performed came from 37 gravid dams (labeled D1 to D40) that were acquired from a French rabbit farm for which the antibiotic history was available. The dams were selected so that they all received diclazuril (coccidiostat), tilmicosin (macrolide), sulfadimethoxine (sulfonamide), oxytetracycline and trimethoprim. They also received a subcutaneous injection of 23 mg/kg of sulfadimethoxine and 5 mg/kg trimethoprim twice a day for three days and 100 mg/kg oxytetracycline by a single subcutaneous injection 25 days before their due date. They arrived in the animal facility of the French National Institute for Agricultural Research (INRA) 18 days before their parturition. In contrast, the three non-gravid “donor” does (D41, D43 and D44) producing the competitive microbial communities were from farms that have banned the use of antibiotics for at least 5 years.

### Suspensions of the Competitive Microbial Communities From Source Does

The fecal suspensions tested for competitive exclusion were prepared less than 2 h before their oral administration: for each donor doe, five fecal pellets (approx. 5 g) were added to a bottle containing 5 g of 1-mm glass beads and 40 mL 0.9% NaCl. After the bottle was sealed with a septum, oxygen was purged through a 0.1-mm needle via five cycles of pumping at −700 mbar and filling with 500 mbar N_2_. The content was then homogenized using vigorous shaking.

### Growth Environment of the Animals, Competitive Microbial Exposure and Sampling

Gravid dams were fed with a standard diet (Aliment Lactation, Hycole, France) until weaning. They were raised in cages measuring 100 × 42 × 60 cm that included 24 × 42 cm nests that could be closed by a sliding door. Suckling was allowed from 8 to 8:30 am for the first 21 days. From Day 1 to 21, dams had no access to their kits other than during the suckling window, which is common practice until Day 7 for meat rabbit farming in France. The litters were equilibrated to 6–8 kits per litter at Day 1. The 37 dams that had already given birth (Day 0) were divided into eight groups. The kits in the control group were raised as usual; the kits from the “No Feces” control group (i.e., “Control NF”) were raised as usual except that we removed all maternal fecal pellets after suckling up to Day 21. For the kits of the six treated groups, we also removed the maternal fecal pellets after suckling but we put them in contact with the fecal microbes of a “donor” Doe (D41, D43 or D44) until Day 29. Each donor doe was kept individually in cages located in different rooms for the whole duration of the experiment. The groups I41, I43, and I44 (Inoculation from D41, D43, and D44, respectively) received the microbial suspensions prepared as described above by letting them suck 100 μl from a syringe every second day between 10 and 11 am from Day 2 to 29. Gloves and syringes were changed between each cage to avoid cross-contamination. For the groups P41, P43, and P44 (fecal pellets from D41, D43, and D44, respectively), we put five fresh fecal pellets from the relevant donor doe in the nest containing the kits. At Day 21, the nests were opened and kits could freely roam in the whole cage and interact with the dam until Day 36. At Day 35, one kit per cage was anesthetized via an intramuscular injection of 80 mg ketamine/kg (Chlorketam 1000, Vétoquinol, France) followed by an intracardiac injection of 182 mg pentobarbital/kg (Dolethal, Vétoquinol, France), causing death. The experiments were conducted under the agreement number APAFIS-2018021215568441 for animal experimentation from the French Ministry of Agriculture (Ethics Committee C2EA-86). The fecal pellets were immediately recovered from the digestive tract and a 100-mg subsample was placed in dry ice and then stored at −80°C for DNA extraction. Another 500-mg subsample was mixed with 3 mL of sterile phosphate-buffered peptone water containing 30% w/w glycerol and immediately mixed with a pestle by hand. The resulting homogenate was plated on MacConkey medium to isolate *Enterobacteriaceae*, and 10 colonies per sample were stored at −80°C in order to later test their resistance to antibiotics. At Day 3, 17, and 35, fresh fecal pellets from maternal and the three donor does were recovered and placed in dry ice. A 2-g subsample was homogenized with a bagmixer (90 s, setting 8) for isolation and storage of *Enterobacteriaceae*, as previously described for kit fecal samples. At Day 35, a fecal pellet from each doe was immediately stored at −80°C for DNA extraction.

#### Testing Antibiotic Resistance of *Enterobacteriaceae* Isolates

The antibiotic susceptibility of *Enterobacteriaceae* isolates collected from the does or the kits was tested by plating a 24-h pure culture onto Mueller-Hilton agar supplemented with 16 μg/mL tetracycline or 128 μg/mL sulfamethoxazole according to the ECOFF *E. coli* epidemiological cut-off values defined by Eucast (eucast, 14/05/2015). Each test included a resistant and sensitive control strain to confirm that the antibiotic was active and that the incubation conditions allowed growth. The average percentage of antibiotic-resistant *Enterobacteriaceae* isolates was calculated for each lactating doe (maximum of 30 isolates with 10 isolates per sampling day) and for their nest (maximum of 30 isolates with 10 isolates for one kit per nest). When the number of isolates was too low to calculate an average percentage (*n* < 10 for the doe or the nest) and/or when the average percentage of antibiotic-resistant *Enterobacteriaceae* of the lactating doe was less than 50%, we excluded the maternal doe/kit couple from the analysis.

### DNA Extraction and Detection of Antibiotic Resistance Genes

The microbial DNA was extracted at weaning from the three donors, the 37 dams and the 37 kits using the ZR-96 Fungal/Bacterial DNA Kit^TM^ in which 80 mg of frozen feces were processed according to the manufacturer’s instructions (Zymo Research Corp, Irvine, CA, United States). The microbial DNA was also extracted from the three donor does at 16 days for ARG detection. The DNA concentration was measured using the Quant-iT TM PicoGreen TM dsDNA Assay Kit according to the manufacturer’s protocol (Invitrogen, Carlsbad, CA, United States) and subsequently diluted to 10 ng/μl.

High throughput real-time qPCR was performed using the Biomark microfluidic system from Fluidigm (San Francisco, CA, United States) using a 96.96 Dynamic Array^TM^ Integrated Fluidic Circuit (IFC). Pre-amplification of the samples, chip loading and qPCR reactions in nanoliter volumes were performed according to the manufacturer’s protocol. A pre-amplification step was applied to all samples for all primer sets except the *Zhu16S* primer set. Briefly, 13 ng of total DNA were submitted to 14 PCR cycles using the PreAmp Master Mix (Fluidigm) and a mix of primers (50 nM final concentration). Pre-amplified samples were 5-fold diluted after an exonuclease treatment. The diluted pre-amplified samples and the primer sets were loaded in a 96.96 IFC using an IFC Controller HX (Fluidigm). The Biomark thermal protocol was as follows: a thermal mix step (50°C, 2 min; 70°C, 30 min; 25°C, 10 min), a hot start (50°C, 2 min; 95°C, 10 min), 35 cycles of PCR (95°C, 15 s; 60°C, 60 s), and a final melting phase (60°C to 95°C). The list of primers can be found in Supplementary Table S1. The relative quantities for each gene was interpolated using a generated standard curve build from serial dilutions with Fluidigm real-time PCR analysis software (v4.3.1) Supplementary Figure S9 and compared to the negative control. The abundances of each ARG relative to the 16S rRNA genes were then used for further analysis.

### Microbial Community Analysis by 16S rRNA Gene Sequencing

The V3V4 region was amplified from the 77 purified genomic DNA with the primers F343 and R784 (Supplementary Table S1) using 30 amplification cycles with an annealing temperature of 65°C. Because MiSeq (chemistry v3) enables paired 250-bp reads, the ends of each read are overlapped and can be stitched together to generate extremely high-quality, full-length reads of the entire V3 and V4 region in a single run. Single multiplexing was performed using 6 bp indexes developed in-house, which were added to R784 during a second PCR with 12 cycles using indexing forward primer and reverse primer. The resulting PCR products were purified and loaded onto the Illumina MiSeq cartridge according to the manufacturer’s instructions. The quality of the run was checked internally using PhiX, and then each pair-end sequence was assigned to its sample with the help of the previously integrated index. Each pair-end sequence was assembled using Flash software (Magoc and Salzberg, 2011) with an overlap of at least 10 bp between the forward and reverse sequences, allowing 10% of mismatch. The lack of contamination was checked with a negative control during the PCR (water was used as the template). The quality of the stitching procedure was controlled using four bacterial samples that are run routinely in the sequencing facility in parallel to the current samples. The resulting sequences were stored in Genbank (Bioproject SRP100061). Sequences longer than 300 bp were checked for chimeras and clusterized at 0.03 and taxonomically assigned with USEARCH v8.1.1861 (Edgar, 2013) using rdp trainset 16, yielding 15334 ± 3018 sequences per sample. The table was rarefied with the R package phyloseq1.16.2 (McMurdie and Holmes, 2013).

### Statistical Analysis

The statistical analysis was performed with R 3.3.1 (R Development Core Team, 2008). The significance between the relative abundances of antibiotic-resistant genes in lactating dams vs. donor does was tested using the Mann–Whitney–Wilcoxon test for two groups with the Benjamini–Hochberg correction for multiple testing. This was performed with the wilcox.test and the p.adjust functions. The significant confidence level was set at 0.05.

The difference in the abundances of ARGs between the dams in the eight groups was tested using the Kruskal–Wallis test with the Benjamini–Hochberg correction of the two-tailed Dunn’s test for multiple testing since ARGs could increase or decrease. The efficiency of fecal pellets vs. the corresponding suspension in decreasing ARGs was tested using a paired *t*-test. In other words, the decrease observed in the fecal pellets was compared to the decrease observed in the suspension for each ARG. The difference of the percentage of antibiotic-resistant *Enterobacteriaceae* between the different groups of lactating dams or groups of kits was also tested using the Kruskal-Wallis test, but the multiple testing was performed with the pgirmess package (version 1.6.9) in order to obtain a two-tailed *p*-value since we assume that the percentage of antibiotic-resistant genes decreases.

The richness was estimated by the Chao1 estimate using the estimateR function (R package vegan 2.4-2), which was then analyzed by a one-way analysis of variance (ANOVA) to detect an impact of the group. The significance of the separation observed on the nMDS plot using the Bray-Curtis dissimilarity was tested using pairwise permutational multivariate analysis of variance with distance matrices on the square-root-transformed abundances of the operational taxonomic units (OTUs) with the adonis function, after confirming the homogeneity of the variance though the betadisper function (R package vegan 2.4-2). Two samples out of 37 were ignored because of aberrant profiles (kit10 and kit28).

In order to quantify the immigration rates under the assumptions of the neutral model, we used the script provided by Burns et al. (2016).

The linear and the non-linear relationships between the OTUs and the ARGs were characterized by the maximal information coefficient (MIC) index (Reshef et al., 2011). Briefly, this index compares a *X*- and an *Y*- vector of values by quantifying the non-randomness of points in a two-dimensional space. This index was calculated on all the OTUs with more than 10 counts on average, which represented 104 OTUs. A relationship was considered significant if its MIC value was observed in less than 1% of the 37950 MIC values across 100 randomized datasets. In our case, the MIC threshold values were 0.66365 for the kits and 0.65429 for the dams.

## Results

### Comparison of the ARGs in the Feces From the Lactating Dams and the Donor Does

As expected, many antibiotic resistance genes are reduced in does from farms that have banned antibiotics for several years (referred to as “donor” does below). Based on the 37 samples from the lactating dams and the six samples from the donor does, half of the ARGs (15 out of 26, Supplementary Table S2) are significantly reduced if we consider the three donor does as a group, despite their heterogeneity in ARGs. Most of the ARGs (21 out of 26, Table 1) were still detectable in the feces of all the donor does, suggesting that ARGs might persist for years even in an antibiotic-free environment.

**TABLE 1:** Ratios of relative ARGs abundances in kits and in the three donor does.

		**ARG ratio of kits vs. control kits (*n* = 37 litters, one kit per litter)**							**ARG ratio of donor does vs. lactating dams (*n* = 37)**		
	**ARG**	**ControlNF/ Control**	**I41/ Control**	**P41/ Control**	**I43/ Control**	**P43/ Control**	**I44/ Control**	**P44/ Control**	**donor Doe41/Lactating Dams**	**donor Doe43/Lactating Dams**	**donor Doe44/Lactating Dams**
Aminoglycoside	*aac6Im*				0.38			0.4	0.47	0.16	0.11
	*aacA.aphD*								0.15	0.00	0.01
	*aadE*				0.45	0.49			0.57	0.29	0.51
	*ant6Ib*					0.42			0.44	0.23	0.16
	*aph2Ib*				0.40	0.28		0.4	0.51	0.06	0.13
	*aph3Ib*								2.10	0.05	0.02
	*aphA3*				0.49	0.32			0.44	0.31	0.31
	*MGaph*								0.03	0.02	0.00
	*strB*								2.40	0.02	0.00
Beta lactam	*CblA1*				0.04			0.04	0.11	0.19	0.04
	*cepA29*								2.25	0.26	0.01
Macrolide	*ermB*				0.47	0.19			0.43	0.09	0.03
	*ermG*				0.21	0.11	0.5		0.18	0.08	0.13
	*lnuC*								1.01	0.38	0.12
	*mefA*		311						15.70	1.05	0.01
Phenicol	*floR*								1.73	2.46	0.15
Sulfamide	*sul2*								48.50	0.00	0.00
Tetracycline	*tet32*		1.7						0.46	0.31	1.10
	*tet33*				9.20			11.8	4.82	1.31	0.08
	*tet40_1*				0.45	0.32			0.63	0.44	0.27
	*tetM*								0.84	0.15	0.70
	*tetO*								0.35	0.86	0.18
	*tetQ*								0.32	0.63	0.37
	*tetY*								0.00	0.00	0.00
Trimethoprim	*dfrD*								0.88	0.00	0.00
Vancomycin	*vanTG*								0.84	1.34	2.27
Significance of	*p*-values	0.62	0.5	0.2	0.08	0.02^*^	0.2	0.02^*^			
separation of the communities from the controls based on 16S data	*R*^2^	0.12	0.10	0.14	0.13	0.16	0.12	0.16			

The kits are compared to the kits of the control group at weaning. The donor does are compared to the lactating dams. The value of ARGs in the donor does was highlighted when it was lower than the ARG abundance in the lactating dams. The ratios are reported only when the kits exposed to the microbial communities were significantly different from the control kits. The ratios higher than 1 are in red (see Supplementary Table S3 for values before ratio). The pairwise *p*-values of the group separation of the microbial communities from the controls based on 16S rRNA sequences are also reported. ^*^Indicates statistical significance.

### Both Inoculation of Suspensions and Contact With Fecal Pellets Decrease the Total Carriage of Antibiotic-Resistant Genes in Kit Gut Microbiota

When inoculating the kits with a microbial suspension every second day between day 2 and 29, suspension I43 reduced eight antibiotic resistance genes (*aac6Im*, *aadE*, *aph2Ib*, *aphA3*, *CblA1*, *ermB*, *ermG*, and *tet40*), whereas suspension I44 reduced the abundance of *ermG* by half (Table 1). It should be noted that the inoculation can also add an antibiotic resistance gene if that gene is abundant in the donor doe. For example, donor Doe41 exhibited an unexpectedly high amount of *mefA* (macrolide efflux pump), which explains why kits fed with suspension I41 had 312-fold more *mefA* than the control kits.

Adding the fecal pellets to the nest is slightly more efficient than oral ingestion to drive the competitive exclusion. Indeed, six ARGs decreased in both kits exposed to suspension I43 and to the fecal pellets of Doe43, but the decrease was more drastic for kits exposed to pellets (72% vs. 59%, *p* = 0.027). In addition, the fecal pellets from Doe44 decreased the carriage of three different ARGs (*aac6Im*, *aph2Ib*, and *cblA1*), whereas the corresponding suspension only changed *ermG*.

While adding fecal pellets from antibiotic-naive donor does generally decreases the ARG transmission from a dam to its kits, the relative success could not always be simply inferred from the individual levels of the antibiotic resistance genes in the donor does (Table 1 and Supplementary Figure S1). For example, adding fecal pellets of Doe43 decreased *tet40* abundance in the kits by 68%, but adding fecal pellets from Doe44 had no effect, even though the latter had a lower abundance of *tet40* (Table 1). This was confirmed by a second pair of primers targeting *tet40* (0.24 vs. 0.40 arbitrary units, Supplementary Table S2). In other words, the competition does not simply occur between a microbe carrying the ARG and its relative lack thereof.

### Inoculation of Suspensions and Contact With Fecal Pellets Also Decrease the Carriage of Antibiotic-Resistant *Enterobacteri*a

**FIGURE 1:**
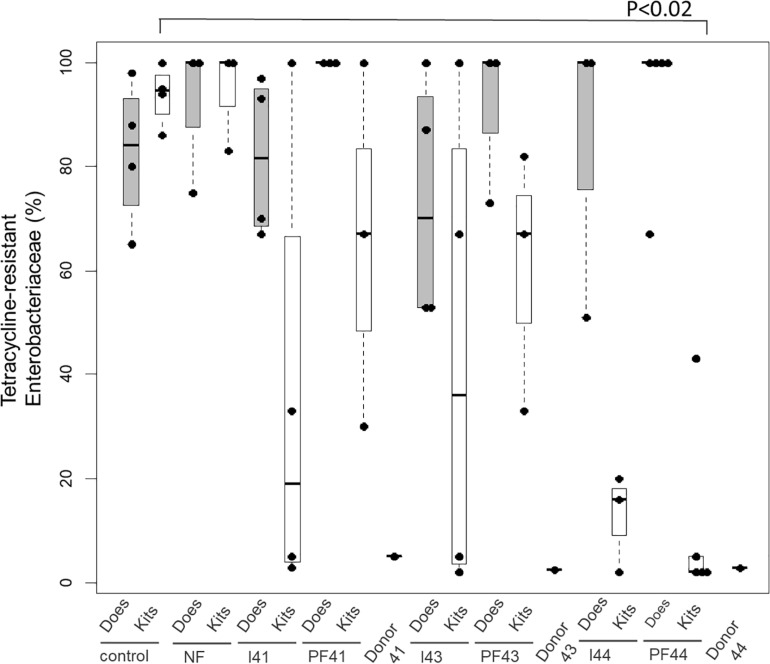
Proportion of *Enterobacteriaceae* isolates resistant to tetracycline in the eight groups: controls (C), the no-feces controls (NF), Inoculum41 (I41) and fecal pellets of Doe41 (P41), Inoculum43 (I43) and fecal pellets of Doe43 (P43), Inoculum44 (I44) and fecal pellets of Doe44 (P44). The gray bars indicate the proportion of resistant *Enterobacteria* in the dams at weaning; the white bars show the resistance in the corresponding kits at weaning. The proportion of *Enterobacteriaceae* isolates in the donor does is also indicated.

The higher efficiency of fecal pellets for competitive exclusion is also suggested when considering the exclusion of *Enterobacteria* resistant to tetracycline estimated from 10 isolates per kit (Figure 1 with four or five kits per group at D35). Indeed, the fecal pellets from Doe44 significantly excluded these bacteria (*p* = 0.02), while suspension I44 shows a trend that only becomes significant when performing a second trial (Supplementary Table S7 and Supplementary Figure S2). In contrast, the ratio of tetracycline-resistant *Enterobacteria* in the “No Feces” controls was similar to the ratio observed in the lactating does, so that removing the feces potentially left by the lactating doe is not enough to stop the transmission of antibiotic-resistant bacteria. It should be noted that 14 out of 59 *Enterobacteriaceae* isolates were resistant to tetracycline in donor Doe41, four out of 50 in Doe 43, whereas no isolates were resistant in Doe44.

### Inoculation of Suspensions and Contact With Fecal Pellets Impact the Total Microbial Community of the Kits

**FIGURE 2:**
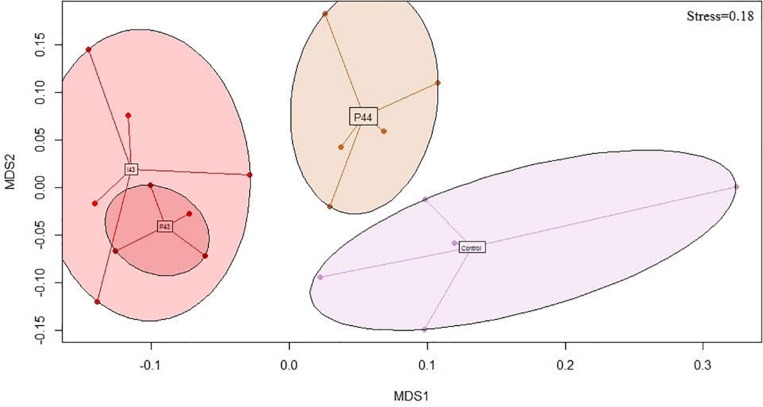
Bray-Curtis nMDS projection of the fecal microbial communities of the kits monitored by sequencing the 16S rRNA genes at weaning (stress = 0.18). Only the groups that tend to be different from the controls (i.e., *p* < 0.1) are represented: P44 shows the kits exposed to the fecal pellets of the donor Doe44; I43 and P43 show the kits exposed to the microbial suspensions and the pellets of the donor Doe43, respectively; the control kits are kits raised in standard conditions.

We generated 1.4 M of 16S rRNA sequences across 4023 OTUs after rarefaction at 5423 sequences per sample. The richness was not impacted (681 ± 145 OTUs) but the fecal communities of the kits in contact with fecal pellets from Doe43 and Doe44 differed from the communities observed in the control kits, unlike the fecal communities of kits in contact with fecal pellets from Doe41 (*p*-values from ADONIS = 0.004, 0.008, and 0.3, respectively, Table 1). This impact is illustrated by the non-metric dimensional scaling (nMDS) representation, which shows the similarity of the impact of the oral ingestion of the suspension vs. adding the fecal pellets in the nest on microbial communities (Figure 2 and Supplementary Figure S3). The microbiota of the lactating does differed from the microbiota of the kits (58% of the OTUs detected in kits were absent from the does, Supplementary Figure S4) but reassuringly did not present a group pattern similar to the pattern observed in the kits (*p* = 0.8, Supplementary Figure S5), which confirms that the separation between the groups is due to the inoculations. As observed for the transmission of ARGs and *Enterobacteria*, simply removing the feces potentially left by the lactating mother had no significant impact on the microbiota of the kits at weaning (*p* = 0.62, Table 1). Consistently with the lack of effect of microbes from Doe41 described above, Suspension I41 and the corresponding fecal pellets also had no significant impact on the microbial community of the weaning (*p* = 0.7 and 0.3, Table 1). The abundances of the OTUs in the microbial communities of the 37 kits were not directly correlated to their abundance in the microbiota of the corresponding 37 does, even when keeping the mother-kit couple (Supplementary Figure S4). Still, the metacommunity can be estimated from the measurements in the kits within each group. Interestingly, this species abundance distribution revealed that the more immigration there was from the metacommunity, the stronger the impact on the total microbial community of the kits would be. Indeed, the *p*-value of the impact on the total community correlates negatively to the immigration rate predicted by the neutral model using the distribution of the OTUs across the samples in relation to their relative abundance (*r*^2^= 0.4, Supplementary Table S4 and Supplementary Figure S6).

### Identification of Clusters of ARGs

**FIGURE 3:**
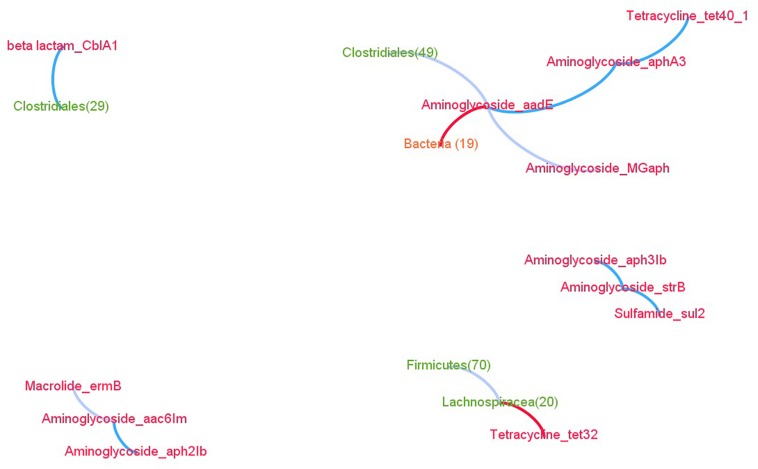
Network of the significant linear (blue edges) and non-coexistence (red edges) associations between ARGs (red nodes) and OTUs. Unclassified associations are in gray.

By comparing the abundance of the ARGs across the kits, we identified three significant ARG clusters, i.e., ARGs whose abundance are more correlated than chance would predict (Supplementary Table S5 and Supplementary Figure S7): *aph2Ib*/*aac6Im*, *aph3Ib*/*strB*/*sul2*, and *MGaph*/*aadE*/*aphA3*/*tet40* (Figure 3). Interestingly, the latter two contain genes from different classes of antibiotics (namely aminoglycoside and sulfamide or aminoglycoside and tetracycline). Naturally, the abundance of amplicons obtained from primers targeting the same gene also fall into the same clusters, such as *tet40_1* and *tet40_2*. Despite the marked differences in microbiota composition between kits and dams, the *aph2Ib*/*aac6Im* and *aph3Ib*/*strB* clusters are also observed in the dams (Supplementary Table S6 and Supplementary Figure S8).

### Identification of OTUs That Are Associated With ARGs

In kits, four OTUs were significantly associated with the ARGs. First, *CblA1* was proportional to OTU26, affiliated with the *Clostridiales* order and commonly observed in rabbits (Ley et al., 2008). Second, *MGaph* did not coexist with OTU19 and had a complex relationship with OTU49 (*Clostridiales*). Finally, *tet32* did not coexist with OTU20 belonging to the *Lachnospiraceae* family (Figure 3 and Supplementary Table S4).

In does that were analyzed separately to avoid spurious relationships (Supplementary Table S6), the only significant ARG-OTU relationships were *CblA1* being proportional to OTU21, affiliated with the *Bacteroides* genus in which this gene was first described [20], and *ermB* (target methylation) associated with OTU95 belonging to the *Clostridales* order in which *ermB* determinants were previously detected in *Clostridium* (Spigaglia et al., 2005) and in *Ruminococcus* (Kennedy et al., 2018). It should be noted that the closest affiliation of OTU95 was *Ruminococcus*, albeit with a low confidence (0.26).

## Discussion

### Evidence for Vertical Transmission

In our study, we modified the microbial community that colonizes the gut of the kits using microbial fecal communities from foreign does by either providing them as anaerobic suspensions or as fecal pellets in the nest before weaning. The “donor” does are from farms that have banned antibiotics for at least 2 years. The feces are collected from the kits at weaning, at which point 26 antibiotic-resistant genes are evaluated via quantitative PCR, the gene coding for the small ribosomal subunit is sequenced, and the cultivable *Enterobacteriaceae* are tested for tetracycline and sulfonamide resistance using classic culturing techniques.

The vertical transmission of antibiotic-resistant microbes from mother to kits is confirmed by the control group in which the ratio of antibiotic-resistant *Enterobacteria* in the feces of the kits mirrors the ratio observed in lactating dams. It should be noted that this ratio does not depend on age, unlike the abundance of the OTUs, as previously described in rabbits (Combes et al., 2014), in humans (Odamaki et al., 2016), and in mice (Langille et al., 2014). This intergenerational transmission might be the reason why ARGs against phenicols are still detectable, even though they have not been used in French rabbits for at least 20 years (Anses editor, 2011), and *Streptomyces*, known to produce phenicols, was undetectable in our samples. Luckily the intergenerational transmission of OTUs and the associated ARGs can be interrupted when using the correct inoculum and delivery method, as described below.

### Variability of Ability of the Microbial Communities to Discontinue the Vertical Transmission

The absence of the target is not enough to guarantee a successful exclusion. For example, Doe41 had a low abundance of resistant *Enterobacteria*, yet fails to exclude them. For ARGs as well, the mere absence of the ARG to be outcompeted is not sufficient. For example, Doe43 has more copies of *ermB* than Doe44, yet, paradoxically, only the microbes from Doe43 decrease the carriage of *ermB* in the kits. Likewise, Doe43 and Doe44 are both free from tetracycline-resistant *E. coli* according to our detection method, yet only microbes from Doe44 significantly decrease the proportion of tetracycline-resistant *Enterobacteria* (*p* = 0.02). Predicting the exclusion of targeted genes from complex communities is a critical milestone that requires additional information about the microbiota performing the exclusion. Indeed, it is challenging to predict the outcome of the exclusion based on the abundance of the ARGs in the donor doe and the lactating dam (Supplementary Figure S1). It should be noted that the outcompeting microbes are not necessarily ARG-free variants that are phylogenetically close: for example, vancomycin-resistant *Enterococcus* belonging to the *Proteobacteria* phylum are outcompeted *in vivo* by the *Barnesiella* genus belonging to the *Bacteroides* (Ubeda et al., 2013). Interspecific exclusion could explain why ARGs increase or decrease in a surprising manner, as mentionned above. Such an interspecific exclusion also implies that exclusion cannot be efficiently understood by investigating the species of interest alone but requires data about the whole microbial community. Indeed, simply replacing the targeted antibiotic-resistant microbes by their antibiotic-sensitive conspecifics is temporary at best. In pigs for example, using antibiotic-sensitive *Megasphaera* only delays the appearance of antibiotic-resistant strains (Stanton and Humphrey, 2011), possibly because of horizontal gene transfer toward the sensitive *Megasphaera*. By acting on the whole community of the gut microbes at once, exclusion with complex communities hinders the vertical transmission of microbes as well as the horizontal transfers of ARGs from ARG-rich communities toward the sensitive microbes, which might occur between microbial species via plasmids, transposons or even phages (Muniesa et al., 2013; Zhu et al., 2013).

### Impact of the Mode of Delivery of the Microbial Communities on the Success of Competitive Exclusion

The success of competitive exclusion also depends on the delivery method of the microbes: adding fecal pellets was more efficient than anaerobic suspensions. This is surprising because oxygen may penetrate the fecal pellets, thereby killing 73% of the bacteria, which are primarily anaerobic in the gut (Harris et al., 1976) within minutes (Brusa et al., 1989). Furthermore, competitive experiments confirmed that microbial communities are more likely to contain bacteria excluding the targeted species when they are kept in anaerobic conditions (Ubeda et al., 2013). In contrast, other authors suggest that oxygen exposure could be less crucial than previously thought. Some bacteria form oxygen-tolerant spores specialized for host-to-host transmission, and even non-spore-forming bacteria can remain viable for 2 days upon oxygen exposure (Browne et al., 2016). In addition, oxygen can be used by 23% of the bacteria in the lumen [which are facultative anaerobes (Harris et al., 1976)] and by gut microbes from the mucus (Albenberg et al., 2014), suggesting that oxygen plays a role in the gut. In our experiment, the role of oxygen remains unclear because microbial respiration from the gut bacteria that tolerate oxygen may limit the actual penetration of oxygen in the fecal pellets. Regardless, we demonstrated the relevance of coprophagy for the intergenerational transmission of microbes and associated ARGs.

Interestingly, coprophagy is found in many animal species including pigs (Orland and Brand, 1991), rabbits (Combes et al., 2014), termites (Rosenberg and Zilber-Rosenberg, 2011), horses and rats (Galef, 1979; Crowelldavis and Houpt, 1985). For example, piglets reportedly ingest 20 g of their mother’s feces daily (Orland and Brand, 1991). Regarding rabbits, the doe defecates in the nest (Kovacs et al., 2006), and the fecal pellets that the kits ingest when they are between 8 and 20 days old change their microbiota at weaning (Combes et al., 2014). This natural behavior probably plays a role in the colonization since when 24-h-old kits are placed in another nest, the colonization of the kit’s gut from the neonatal environment provided by the lactating doe overrides the one from the biological mother (Abecia et al., 2007). In contrast, humans do not have this behavior, which might explain why the birth imprint is more important in humans. Indeed the microbiota composition and the occurrence of resistance genes of newborns are linked to the microbes in the vaginal fluids (Dominguez-Bello et al., 2010, 2016; Alicea-Serrano et al., 2012).

However, coprophagy is not the only force driving the microbial colonization of the gut since removing the fecal pellets had no detectable effect when no foreign microbes were added to the nest. Hence, simply removing the maternal pellets is not enough to fight against the transmission of antibiotic-resistant microbes because the contact during suckling is sufficient to insure transmission of the maternal microbes. This could be due to either the microbial contact during the milking process (which lasts 10 min in rabbits) or to small amounts of bacteria transmitted before birth, as observed in human (Moles et al., 2013). Unfortunately we did not measure the microbiota at birth so we cannot decipher between the two hypothesis. We also did not observe the artificially increased diversity that animals in incubators from day 2 after birth might exhibit (Schmidt et al., 2011), confirming that we are closer to the real conditions than the incubator model in which one single inoculation event was sufficient to drive the microbial communities. Together, these results show that coprophagy overrides the other factors behind the microbial communities in kit guts.

From an applied point of view, outcompeting the maternal microbiota by adding foreign fecal pellets could have broad implications at the farm and at the national level. At the farm level, a “donor” doe could be kept free from any antibiotic treatment in order to use its fecal pellets to reintroduce antibiotic-sensitive microbes to the rest of the herd when a disease outbreak requires antibiotic use in does. While completely eliminating the ARGs does not seem possible, competitive exclusion could also be interesting at the country level for specific resistances. Indeed, Finland has less microbes that hydrolyze third-generation cephalosporins than Sweden, even though they have a similar organization for broilers. The main difference is that Finland used a commercially available complex microbial community excluding *Salmonella* for decades, which probably also decreased the carriage of *E. coli* producing extended spectrum beta-lactamase as a side-effect (Nuotio et al., 2013), suggesting that wide use of competitive exclusion can impact the ARGs at the national level. In this context, clusters of ARGs such as *ant6lb*/*ermB* could be defined as a priority target since many clusters of ARGs were observed in livestock (Johnson et al., 2016). It should be noted that widely applying competitive exclusion might generate a normalization of the microbial community in livestock, as was observed in patients exposed to fecal microbiota transplantation via oral ingestion of lyophilized microbes (Staley et al., 2017).

There are four limitations of our study: firstly, kits were allowed to roam freely in the area of the dam from Day 21 to 36 in order to mimic the real farming conditions. This contact could have blunted the impacts of the treatments observed at Day 36. Secondly, we sampled only one kit in each 37 litters, which was sufficient to observe that the variability within litter was less than the variability between the treatments, but we could not estimate the exact within-litter variability. Thirdly, 2 out of 3 donor does had unexpected high levels in some ARGs, such as *mefA* for example, possibly because the donor does received a diet favoring a bacteria carrying *mefA* but the exact reasons underpinning the high abundance of *mefA* remains unknown. Whatever the cause, this limits the usefulness of these donors to fight against ARGs unless increasing the abundance of *mefA* is acceptable, and a careful assessment should determine the abundance of the ARGs in the donor does even if the donors were not exposed to antibiotics. Fourthly, we rarefied at 5423 sequences per sample, so the rare microbes were not taken into account in the analysis.

## Conclusion

In conclusion, our work demonstrates the transmission of ARGs from the lactating does to their kits and paves the way toward a microbiome-based remediation strategy against ARGs in rabbits.

## Ethics Statement

This study was carried out in accordance with the recommendations of the Ethic Committee C2EA-86. The experiments were conducted under the agreement number APAFIS-2018021215568441 for animal experimentation from the French Ministry of Agriculture (Ethic Committee C2EA-86).

## Author Contributions

OZ, CSA, VD, and AB-M designed the study. CSA and SS extracted the microbial DNA, chose the primers, and analyzed the microbial sequences. LC uploaded the sequences. CSA and OZ carried out the statistical tests. VD and NA screened the *Enterobacteriaceae* isolates for antibiotic resistance. VD, CSA, OZ, and AB-M drafted the manuscript. All authors edited and approved the manuscript.

## Conflict of Interest Statement

The authors declare that the research was conducted in the absence of any commercial or financial relationships that could be construed as a potential conflict of interest.
