# Supplementary material for: Variability of the Ability of Complex Microbial Communities to Exclude Microbes Carrying Antibiotic Resistance Genes in Rabbits
Source: Front Microbiol. 2019 Jul 2;10:1503. doi: 10.3389/fmicb.2019.01503 (PMC6615258; doi:10.3389/fmicb.2019.01503)
Supplement: Supplementary file 3 [file Data_Sheet_3.pdf]

Supplementary Material

Variability of the ability of complex microbial communities to exclude microbes carrying antibiotic resistance genes in rabbits

Caroline Achard [1,2,a], Véronique Dupouy [3,a], Suzanne Silviglia[1,3], Nathalie Arpaillange[3], Laurent Cauquil[1], Alain Bousquet-Melou[3] , Olivier Zemb[1*]

* Correspondence:

Olivier Zemb

olivier.zemb@inra.fr

Table of contents

1 Flow chart summarizing the experimental design 3
2 ARGs in the donor does and the lactating dams 3
2.1 Pre-amplification of the ARGs 3
2.2 List of primers (Table S1) 3
2.3 ARGs different in donor does and lactating dams (Table S2) 5
3 Additional visualization of the microbiota based on 16S rRNA genes 6
3.1 Other non-metric dimensional scaling (nMDS) for the microbiota of kits (Fig. S3) 7
3.2 Controlling the absence of any pre-existing pattern in the 37 lactating dams (Fig. S5) 8
4 Additional visualization of the exclusion of the ARGs 9
4.1 ARG in the kits (Table S3) 9
4.2 Challenge of predicting the success of competitive exclusion (Fig. S1) 11
5 Additional details about the neutral model 11
5.1 Rationale of the neutral model 11
5.2 The abundance of the OTU in a kit is not proportional to its abundance in the lactating dam 13
5.3 Parameter of the neutral model (Table S4) 14
5.4 Strong immigration correlates with strong differences in kits (Fig. S6) 15
6 Additional details about the networks 16
6.1 Rationale of separate analysis of kits and dams for the analysis 16
6.2 Determination of the significance values for the MINE relationships 16
6.3 List of relationships in kits (Table S5) 16
6.4 Pairwise representation of the relationships detected in the kits (Fig. S7) 17
6.5 List of relationship in dams (Table S6) 18
6.6 ARG clusters and ARG-OTU relationships in the does (Fig. S8) 18
7 Second trial of competitive exclusion 19
7.1 Description of the second trial to measure ARG exclusion 19
7.2 Raw data of the resistant *Enterobacteria* in both trials 19
7.3 Correspondance of sequencing data 20
7.4 Proportion of resistant *Enterobacteria* in both trials (Fig. S2) 22

1 Flow chart summarizing the experimental design

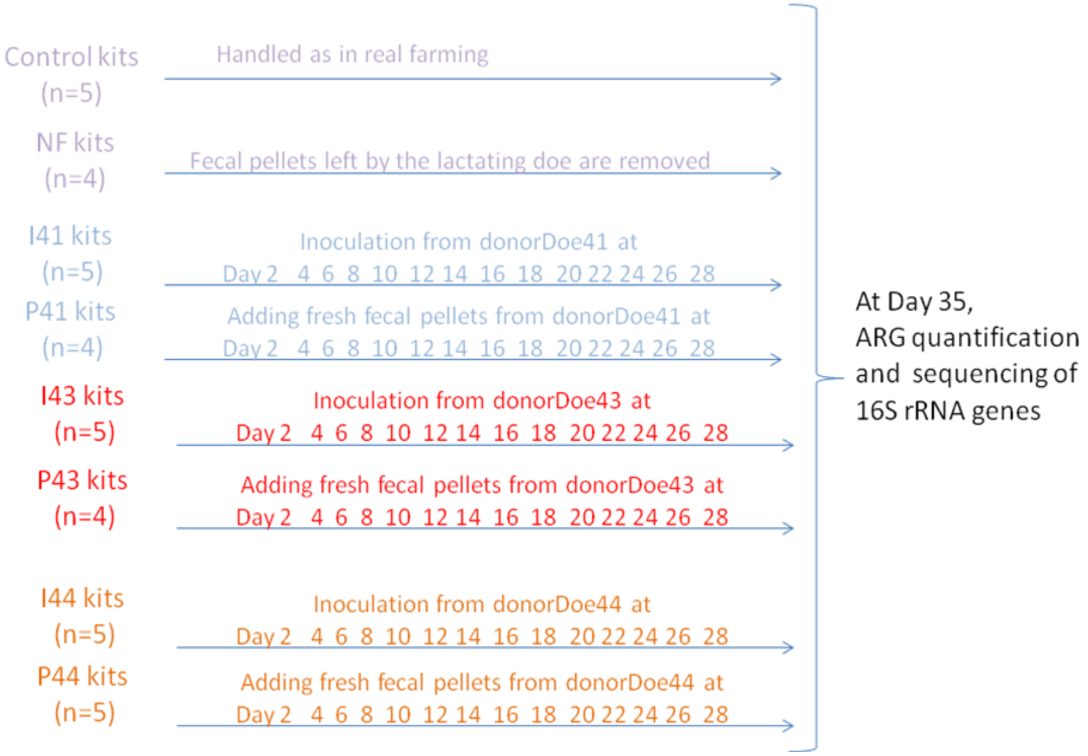

2 ARGs in the donor does and the lactating dams

2.1 Pre-amplification of the ARGs

High throughput real-time qPCR was performed using the Biomark microfluidic system from Fluidigm (San Francisco, CA, USA) using a 96.96 Dynamic Array™ Integrated Fluidic Circuit (IFC). Pre-amplification of the samples, chip loading and qPCR reactions in nanoliter volumes were performed according to the manufacturer's protocol. A pre-amplification step was applied to all samples for all primer sets except the Zhu16S primer set.

Pre-amplification enables amplification of specific DNA targets, and it has been shown to introduce little variability (Korenkova et al., 2015). Such a pre-amplification step is needed for rare ARGs in high throughput methods that use small volumes (Sandberg et al., 2018). It is commonly used to study ARGs in fecal samples (Buelow et al., 2017). It should be noted that the ARG/16S rRNA gene ratios that we obtained are not always between 0 and 1 because of this pre-amplification of ARGs, whereas the 16S rRNA genes were not pre-amplified (see Materials and Methods section).

V. Korenková, J. Scott, V. Novosadová, M. Jindřichová, L. Langerová, D. Švec, M. Šídová, and R. Sjöback, Pre-amplification in the context of high-throughput qPCR gene expression experiment. BMC Molecular Biology 16 (2015) 5

K.D. Sandberg, S. Ishii, and T.M. LaPara, A Microfluidic Quantitative Polymerase Chain Reaction Method for the Simultaneous Analysis of Dozens of Antibiotic Resistance and Heavy Metal Resistance Genes. Environmental Science & Technology Letters 5 (2018) 20-25.

E. Buelow, T.d.j. Bello González, S. Fuentes, W.A.A. de Steenhuijsen Piters, L. Lahti, J.R. Bayjanov, E.A.M. Majoor, J.C. Braat, M.S.M. van Mourik, E.A.N. Oostdijk, R.J.L. Willems, M.J.M. Bonten, M.W.J. van Passel, H. Smidt, and W. van Schaik, Comparative gut microbiota and resistome profiling of intensive care patients receiving selective digestive tract decontamination and healthy subjects. Microbiome 5 (2017) 88.

2.2 List of primers (Table S1)

Table S1: List of 32 primers used in this paper for ARG quantification and sequencing

Class of resistance and ARG name	Primer set	Forward primer	Reverse primer	Reference
Aminoglycoside_aac6Im	*aac6Im*	CGCCCGATGAATGAGGATGA	CACGGTCATCTGTCAGCCAT	This study
Aminoglycoside_aacA.aphD	*aacA.aphD*	AGAGCCTTGGGAAGATGAAGTTT	TTGATCCATACCATAGACTATCTCATCA	(Zhu et al. 2013)
Aminoglycoside_aadE	*aadE*	TACCTTATTGCCCTTGGAAGAGTTA	GGAACTATGTCCCTTTTAATTCTACAATCT	(Zhu et al. 2013)
Aminoglycoside_ant6Ib	*ant6Ib*	ACATCCGACAGCACGTTCTT	CGCAGTAATTCCATACGCACA	This study
Aminoglycoside_aph2Ib	*aph2Ib*	TCAAATCCCTGCGGTAGTGT	CGTCGCTTCATCATATGCAAGG	This study
Aminoglycoside_aph3Ib	*aph3Ib*	GTCAATGGGGCAGCAACTTG	ACTCTTGTCCTCGTCCGGTA	This study
Aminoglycoside_aphA3	*aphA3*	CGGAATTGAAAAAACTGATCGAA	ATACCGGCTGTCCGTCATTT	(Johnson et al. 2016)
Aminoglycoside_MGaph	*MGaph*	GAGAAGGGGAGCATTCGGAG	TTCAGCTTTGTCCCCAGTCC	This study
Aminoglycoside_strB	*strB*	GCTCGGTCGTGAGAACAATCT	CAATTTCGGTCGCCTGGTAGT	(Johnson et al. 2016)
beta lactam_CblA1	*CblA1*	GCCGACGGTATGAAAACTGC	CGTCGGGAAGGATAACGAGG	This study
beta lactam_cepA29	*cepA*	AGTTGCGCAGAACAGTCCTCTT	TCGTATCTTGCCCGTCGATAAT	(Zhu et al. 2013)
Macrolide_ermB	*ermB*	TAAAGGGCATTTAACGACGAAACT	TTTATACCTCTGTTTGTTAGGGAATTGAA	(Zhu et al. 2013)
Macrolide_ermB_2	*ermB_2*	TGAAAGCCATGCGTCTGACA	CCCTAGTGTTCGGTGAATATCCA	(Zhu et al. 2013)
Macrolide_ermB_3	*ermB_3*	CCCGCCATACCACAGATGTT	TGGCGTGTTTCATTGCTTGA	This study
Macrolide_ermG	*ermG*	ACAAACAGATCACTAGCATTGC	GCGCTATCCACTTTAGGTTTTGG	This study
Macrolide_ermG.1	*ermG.1*	ACAAACAGATCACTAGCATTGC	GCGCTATCCACTTTAGGTTTTGG	This study
Macrolide_lnuC	*lnuC*	TTTCTTGATGGTGGCTGGGG	TGGGCTCTTGACTGATATCCA	This study
Macrolide_mefA	*mefA*	CCGTAGCATTGGAACAGCTTTT	AAACGGAGTATAAGAGTGCTGCAA	(Johnson et al. 2016)
Sulfamide_sul2	*sul2*	TCATCTGCCAAACTCGTCGTTA	GTCAAAGAACGCCGCAATGT	(Johnson et al. 2016)
Tetracycline_MGtetM	*MGtetM*	GAAGTGCCGCCAAATCCTTT	CTGCATTCCACTTCCCAACG	This study
Tetracycline_tet32	*tet32*	CCATTACTTCGGACAACGGTAGA	CAATCTCTGTGAGGGCATTTAACA	(Zhu et al. 2013)
Tetracycline_tet32_2	*tet32_2*	GGACACTCCCGGTCATATGG	CACACCGTCTTTTGCCGAAA	This study
Tetracycline_tet33	*tet33*	CGGGATCGTACAGGTTCTCG	TTTGCCTCACCGATCCACTC	This study
Tetracycline_tet40_1	*tet40_1*	TTTGTGCTTGTTGTTGGGGC	TGCCGTTCCTCAATAGCAGG	This study
Tetracycline_tet40_2	*tet40_2*	TGCTATTGAGGAACGGCAGG	ACAAAGCCCAGGTTGAGCTT	This study
Tetracycline_tetM	*tetM*	CATCATAGACACGCCAGGACATAT	CGCCATCTTTTGCAGAAATCA	(Zhu et al. 2013)
Tetracycline_tetO	*tetO*	ATGTGGATACTACAACGCATGAGATT	TGCCTCCACATGATATTTTTCCT	(Johnson et al. 2016)
Tetracycline_tetQ	*tetQ*	CGCCTCAGAAGTAAGTTCATACACTAAG	TCGTTCATGCGGATATTATCAGAAT	(Zhu et al. 2013)
Tetracycline_tetY	*tetY*	CAGGGGCTCTTCTCAATGCA	TTTTGGCTGGCTGTCCTTCA	This study
Trimethoprim_dfrD	*dfrD*	CGGCAAGGATAACGACATTCC	GGTAAAGCCCTTCCGATTGA	This study
Vancomycin_vanTG	*vanTG*	CGTGTAGCCGTTCCGTTCTT	CGGCATTACAGGTATATCTGGAAA	(Zhu et al. 2013)
16S rRNA gene quantification	*X16SZhu*	GGGTTGCGCTCGTTGC	ATGGYTGTCGTCAGCTCGTG	(Zhu et al. 2013)
16S rRNA gene V3V4 sequencing	*F343/R784*	CTTTCCCTACACGACGCTCTTCCGATCTACGGRAGGCAGCAG	GGAGTTCAGACGTGTGCTCTTCCGATCTTACCAGGGTATCTAATCCT	(Drouilhet et al. 2016)

Drouilhet, L., Achard, C.S., Zemb, O., et al. (2016) Direct and correlated responses to selection in two lines of rabbits selected for feed efficiency under ad libitum and restricted feeding: I. Production traits and gut microbiota characteristics. Journal of Animal Science ***94****, 38-48.*

Johnson, T.A., Stedtfeld, R.D., Wang, Q., et al. (2016) Clusters of antibiotic resistance genes enriched together stay together in swine agriculture. MBio ***7****, e02214-02215.*

Zhu, Y.-G., Johnson, T.A., Su, J.-Q., et al. (2013) Diverse and abundant antibiotic resistance genes in Chinese swine farms. Proceedings of the National Academy of Sciences of the United States of America ***110****, 3435-3440.*

2.3 ARGs different in donor does and lactating dams (Table S2)

In order to compare the ARGs in the donor does and in the lactating dams, we quantify the proportion of ARGs with respect to the 16S rRNA gene in each sample. We used the method based on relative standard curve.

Step 1: A standard sample was created to have a positive signal for every ARG and for 16S rRNA gene (which consists in several samples pooled to get all the ARGs so that the matrix of the standard is very similar to the matrix of the samples). This standard sample was then diluted 3 times in 2 fold series.

Step 2: Dilution of the samples originating from each animal (lactating dam, donor dow, or kit).

Step 3: Run the qPCR plate as described in the material and method section.

Step 4: Use the standard curve to obtain the quantities in arbitrary units. This step is also performed by the Fluidigm software. The highest value of the standard is artificially set to 8, while the lowest is set to 1. For each primer pair, the successive dilutions of the standard sample are used to perform a linear regression, which is used to calculate the quantities of *ermB* and 16S rRNA in arbitrary units in a given sample.

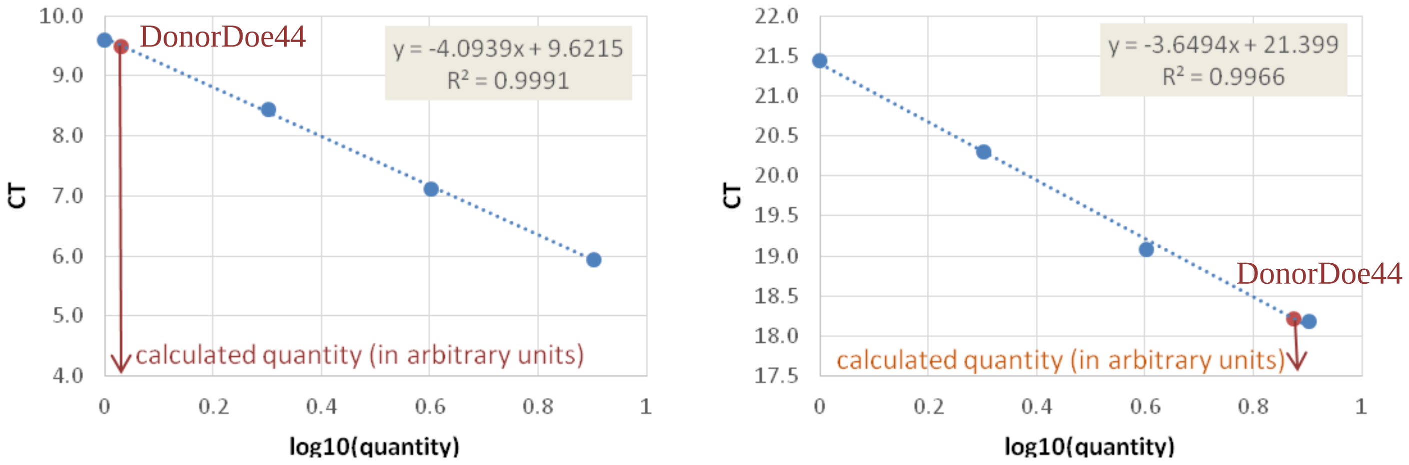

Figure S9: Calculating the quantities in arbitrary units of the *ermB* and 16S rRNA genes for DonorDoe44.

Step 5: Calculate the normalized ARG amount for each ARG by dividing the quantity of each ARG (in arbitrary units) by the quantity of 16S rRNA gene (in arbitrary units). Below we give the example of *ermB*. This step is performed using R software. The normalized amounts are provided in Table S3 below.

$$normalized\_ermB\_in\_DonorDoe44 = \frac{Arbritrary\_ermB\_in\_DonorDoe44}{Arbritrary\_16S\_in\_DonorDoe44}$$

Step 6: Use the normalized ARG amounts (called ARG ratio in Table 1) for the non-parametric analyses to compare the ARG abundances in kits receiving a treatment versus the control kits and the MINE algorithm.

Datasheet1.csv = normalized abundances in does ; Datasheet2.csv = normalized abundances in kits

Step 7: Display the ratio of the donor does vs the lactating dams (Table 1).

$$ermB\_ratio\_DonorDoe44\_vs\_lactating\_dams = \frac{normalized\_ermB\_in\_DonorDoe44}{mean(normalized\_ermB\_in\_lactating\_dams)}$$

Step 8: Use the normalized ARG amounts in the 37 lactating does and in the 3 donor does which have never been exposed to antibiotics to determine the ARGs that are lower in the donors (assuming they are a homogeneous group even though they originate from 3 distinct farms): Out of the 16 significantly affected ARGs, 15 were lower in the donor does (see table S2 below). The *mefA* gene (Line 14) is the exception because it is higher in the donor does than in the lactating dams.

Table S2: ARGs different in donor does and lactating dams. We looked here at the differences in the relative abundances of ARGs between the 37 samples of the lactating dams at weaning and the six samples of the donor does using a Wilcoxon test corrected by Benjamini-Hochberg for multiple testing. Since the technical reproducibility was good, we kept only one primer set per gene for clarity. For each sample, the ARG abundance was normalized with the abundance of the 16S rRNA genes. The values can be higher than 1 because only the ARGs were pre-amplified (see 2.1.).

	ARG	pval_wilcox	p.adj_wilcox	average_in_lactating_dams	average__in_donors
1	Aminoglycoside_aadE	3.28E-07	1.35E-06	1.08168329	0.444526852
2	Aminoglycoside_MGaph	3.28E-07	1.35E-06	0.59857244	0.009318586
3	Macrolide_ermG	3.28E-07	1.35E-06	1.07544853	0.112064662
4	Macrolide_ermB	6.56E-07	2.43E-06	1.08910723	0.139958065
5	Tetracycline_tetQ	2.30E-06	7.72E-06	1.49015581	0.633403019
6	Tetracycline_tet32_2	3.94E-06	1.12E-05	1.36106799	0.535021249
7	Tetracycline_tet40_1	3.94E-06	1.12E-05	0.91289839	0.398649619
8	Aminoglycoside_ant6Ib	2.10E-05	5.18E-05	1.14517676	0.331142995
9	Aminoglycoside_aphA3	4.10E-05	9.48E-05	1.09696471	0.375728162
10	Tetracycline_tetO	7.45E-05	0.00016208	1.1636862	0.447349402
11	Aminoglycoside_aph2Ib	0.00012729	0.00026165	0.74829872	0.171318214
12	Aminoglycoside_aac6Im	0.00026146	0.00050917	0.7921351	0.217765375
13	Tetracycline_tetY	0.00209344	0.00387286	0.00086122	6.89E-05
14	*Macrolide_mefA*	*0.00423166*	*0.00745579*	*0.166842*	*2.335570171*
15	Trimethoprim_dfrD	0.00728886	0.01225853	0.00063822	9.50E-05
16	Aminoglycoside_aacA.aphD	0.0127927	0.02057956	0.00461718	0.000550047
17	Vancomycin_vanTG	0.05952739	0.09177139	1.26501174	1.925139632
18	Aminoglycoside_aph3Ib	0.12979939	0.18471451	0.00104842	0.000777781
19	Aminoglycoside_strB	0.13923405	0.19080222	0.00100063	0.000784073
20	Macrolide_lnuC	0.15957571	0.21086791	3.44595245	1.494660568
21	Sulfamide_sul2	0.29284697	0.37363234	0.00061038	0.007646436
22	Tetracycline_tetM	0.36221089	0.44331309	0.00169957	0.001010005
23	Tetracycline_tet33	0.51664699	0.57927087	0.00342425	0.003963143
24	beta lactam_CblA1	0.57217031	0.62265593	1.36070482	0.375781697
25	phenicol_floR	0.82428507	0.84718188	0.0028028	0.002752934
26	beta lactam_cepA29	0.95914674	0.95914674	1.27828257	1.165971149

3 Additional visualization of the microbiota based on 16S rRNA genes

3.1 Other non-metric dimensional scaling (nMDS) for the microbiota of kits (Fig. S3)

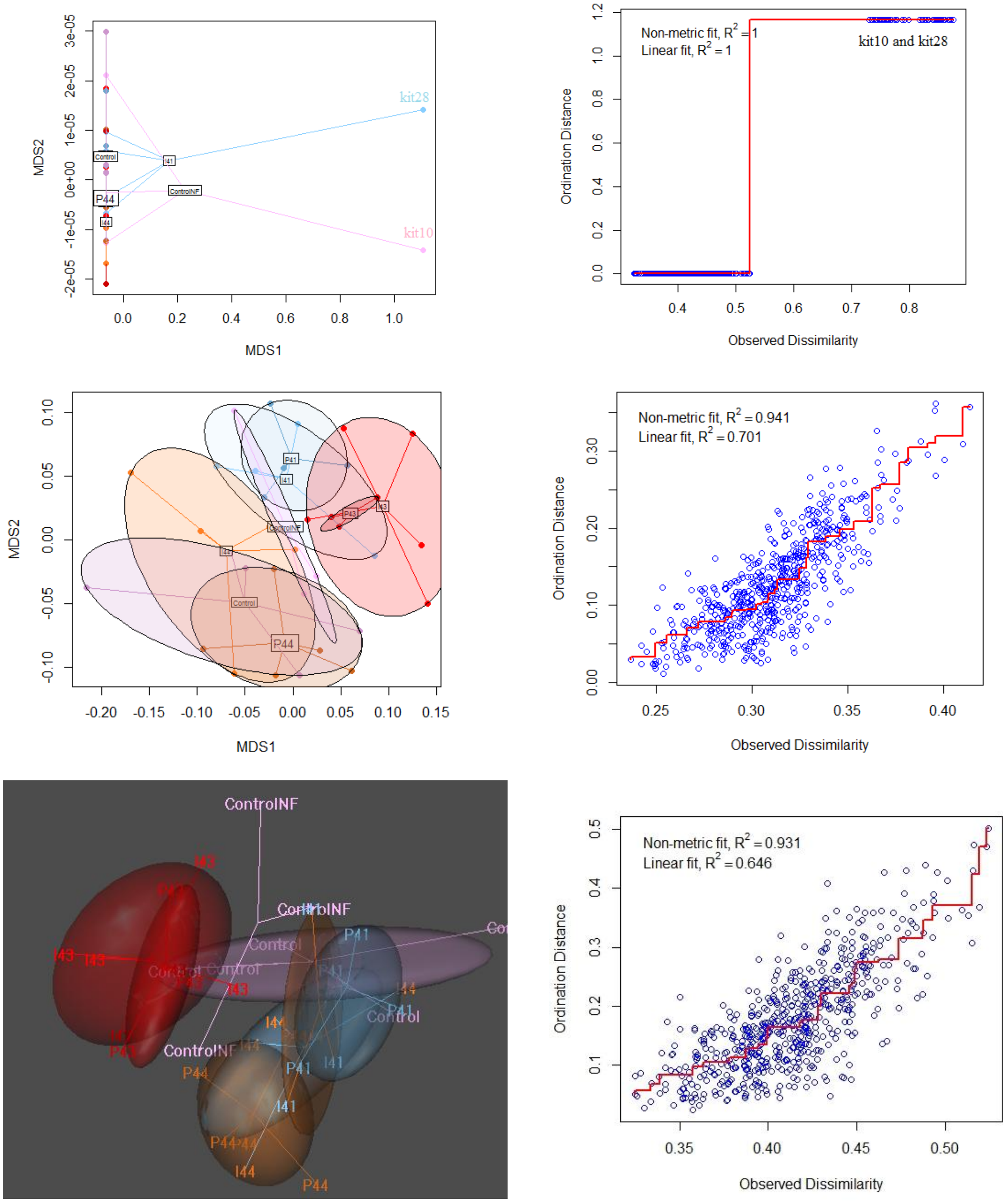

Figure S3: Various representations of the Bray-Curtis nMDS between kit microbiota. The representation with the two outliers is difficult to read (TOP PANEL). The representation with all the samples except the two outliers is easier and the Shepard plot indicates a reasonable fit but the stress remains relatively high even though two convergent solution were found using monoMDS (MIDDLE PANEL, stress=0.24). Finally the 3d-nMDS representation of the gut microbiota from the kits in the eight groups shows a representation of the data with an acceptable stress (stress=0.18) (BOTTOM PANEL). P41, P43 and P44 show the kits exposed to the

fecal pellets; I41, I43 and I44 show the kits exposed to the microbial suspensions prepared from the pellets of donor does D41, D43 and D44, respectively; the control kits are kits raised in standard conditions, whereas the 'ControlNF' are kits raised in the nest from which the maternal fecal pellets were removed.

The pairwise adonis test indicated that the groups P43 and P44 are significantly different from the controls, and that I43 is almost significant (Table S3). The 2D-nMDS projection with all the groups illustrates this, but the stress is relatively high (0.25). The projection below shows that adding an extra dimension decreases the stress but does not drastically change the representation (the stress is 0.19). It simply allows a better discrimination of the P41 and I41 groups.

The graph was obtained with the metaMDS function using three dimensions and the rgl rendering was performed using the vegan3d package, version1.1-2. The ellipses were drawn with a confidence interval of 0.95.

3.2 Controlling the absence of any pre-existing pattern in the 37 lactating dams (Fig. S5)

The lactating dams show no separation pattern of their gut microbial communities according to the permutation test (p=0.8) so that the patterns observed in the kits are not due to a pre-existing difference in the lactating dams, as expected. Since the lactating does are from the same herd, they were placed randomly in the room and the treatments were allocated randomly across the room (therefore, each treatment corresponds to several cages, which are scattered throughout the experimental room).

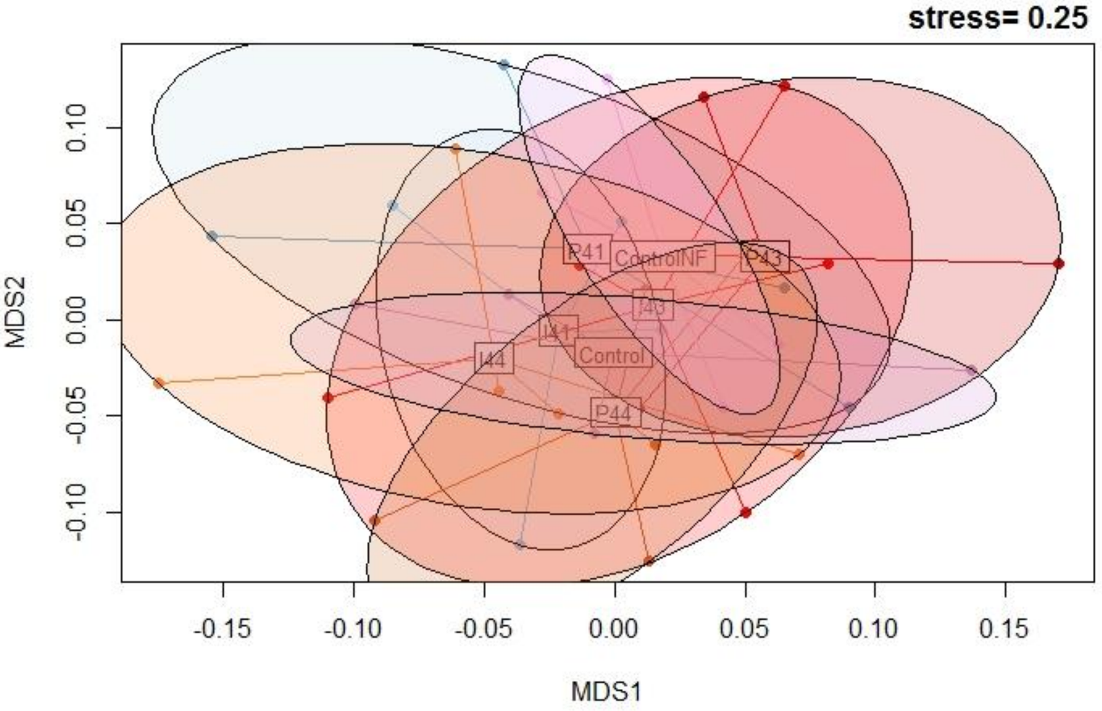

Figure S5: nMDS of the 37 lactating dams. The difference with Fig. 2 is that we analyzed the microbial communities of the dams from each group instead of analyzing the microbial communities of the kits. We used the same method as for the kits (Fig. 2 of the paper), i.e., the Bray-Curtis distance represented with the metaMDS function of the vegan package. The ellipses also indicate the 95% threshold. The absence of a pattern in the dams confirms that the patterns observed in the kits are not due to an unfortunate allocation of the dams.

4 Additional visualization of the exclusion of the ARGs

4.1 ARG in the kits (Table S3)

The ARGs in the kits were measured by qPCR with a pre-amplification step (see Material and Methods section). The table below shows the difference in ARGs between the kits in each group and the control kits. Only significant values are reported. The table also reports the values of each ARG in the lactating dams vs. the donor does. It can be seen that some of the results are intuitive (such as *mefA*, which is abundant in Doe41 and in the kits gavaged with suspension I41). However, some other examples show that there are exceptions to this expectation. For example, *aphA3* has a similar abundance in Doe43 and Doe44, yet only the microbial communities of Doe43 succeed in reducing its abundance in kits. Together, this data indicates that a mere depletion of the targeted ARG is not sufficient to predict its successful exclusion.

Table S3: Abundance of ARGs in kits at weaning and in does, normalized by the abundance of 16sRNA genes in the milking dams and the three donor does. The value of ARGs in the donor does (see section 2.1) was highlighted when it was lower than the ARG abundance in the milking dam. The ratios are reported only when the kits exposed to the microbial communities were significantly different from the control kits. The pairwise ADONIS p-values of the group separation of the microbial communities from the controls based on 16S rRNA sequences are also reported.

		ARG ratio in kits vs. control kits (n=37 litters, one kit per litter)							ARG abundance relative to 16S RNA genes in dams and donor does			
	ARG	ControlNF / Control	I41 /Control	P41 /Control	I43 /Control	P43 /Control	I44 /Control	P44 /Control	milking dams (n=37)	donor Doe41	donor Doe43	donor Doe44
Aminoglycoside	*aac6Im*				0.38			0.4	7.92E-01	3.74E-01	1.27E-01	8.95E-02
	aacA.aphD								4.60E-03	7.00E-04	0.00	4.00E-05
	aadE				0.45	0.49			1.08	6.20E-01	3.09E-01	5.53E-01
	ant6Ib					0.42			1.15	5.10E-01	2.69E-01	1.85E-01
	aph2Ib				0.40	0.28		0.4	7.48E-01	3.81E-01	4.58E-02	9.55E-02
	aph3Ib								1.00E-03	2.10E-03	5.00E-05	2.00E-05
	aphA3				0.49	0.32			1.10	4.82E-01	3.40E-01	3.36E-01
	MGaph								5.99E-01	2.03E-02	1.40E-02	2.88E-03
	strB								1.00E-03	2.40E-03	2.00E-05	0.00
Beta lactam	*CblA1*				0.04			0.04	1.36	1.49E-01	2.63E-01	5.98E-02
	cepA29								1.28	2.88	3.27E-01	1.06E-02
Macrolide	*ermB*				0.47	0.19			1.09	4.71E-01	1.01E-01	3.21E-02
	ermG				0.21	0.11	0.5		1.08	1.91E-01	8.62E-02	1.37E-01
	lnuC								3.45	3.50	1.32	4.29E-01
	mefA		311						1.67E-01	2.63	1.76E-01	1.28E-03
Phenicol	*floR*								2.80E-03	4.83E-03	6.89E-03	4.30E-04
Sulfamide	*sul2*								6.00E-04	2.91E-02	0.00	0.00
Tetracycline	*tet32*		1.7						1.02	4.66E-01	3.13E-01	1.12
	tet33				9.20			11.8	3.40E-03	1.64E-02	4.45E-03	2.70E-04
	tet40_1				0.45	0.32			9.13E-01	5.76E-01	4.01E-01	2.46E-01
	tetM								1.70E-03	1.42E-03	2.60E-04	1.19E-03
	tetO								1.16	4.09E-01	9.93E-01	2.04E-01
	tetQ								1.49	4.74E-01	9.31E-01	5.51E-01
	tetY								8.00E-04	0.00	0.00	0.00
Trimethoprim	*dfrD*								6.00E-04	5.30E-04	0.00	0.00
Vancomycin	*vanTG*								1.27	1.07	1.70	2.88
Significance of separation of the communities from the controls based on 16S data	ADONIS p-values	0.62	0.5	0.2	0.08	0.02 *	0.2	0.02 *				
	ADONIS R^2	0.12	0.10	0.14	0.13	0.16	0.12	0.16				

4.2 Challenge of predicting the success of competitive exclusion (Fig. S1)

The figure below indicates that a mere depletion of the targeted ARG is not sufficient to predict its successful exclusion.

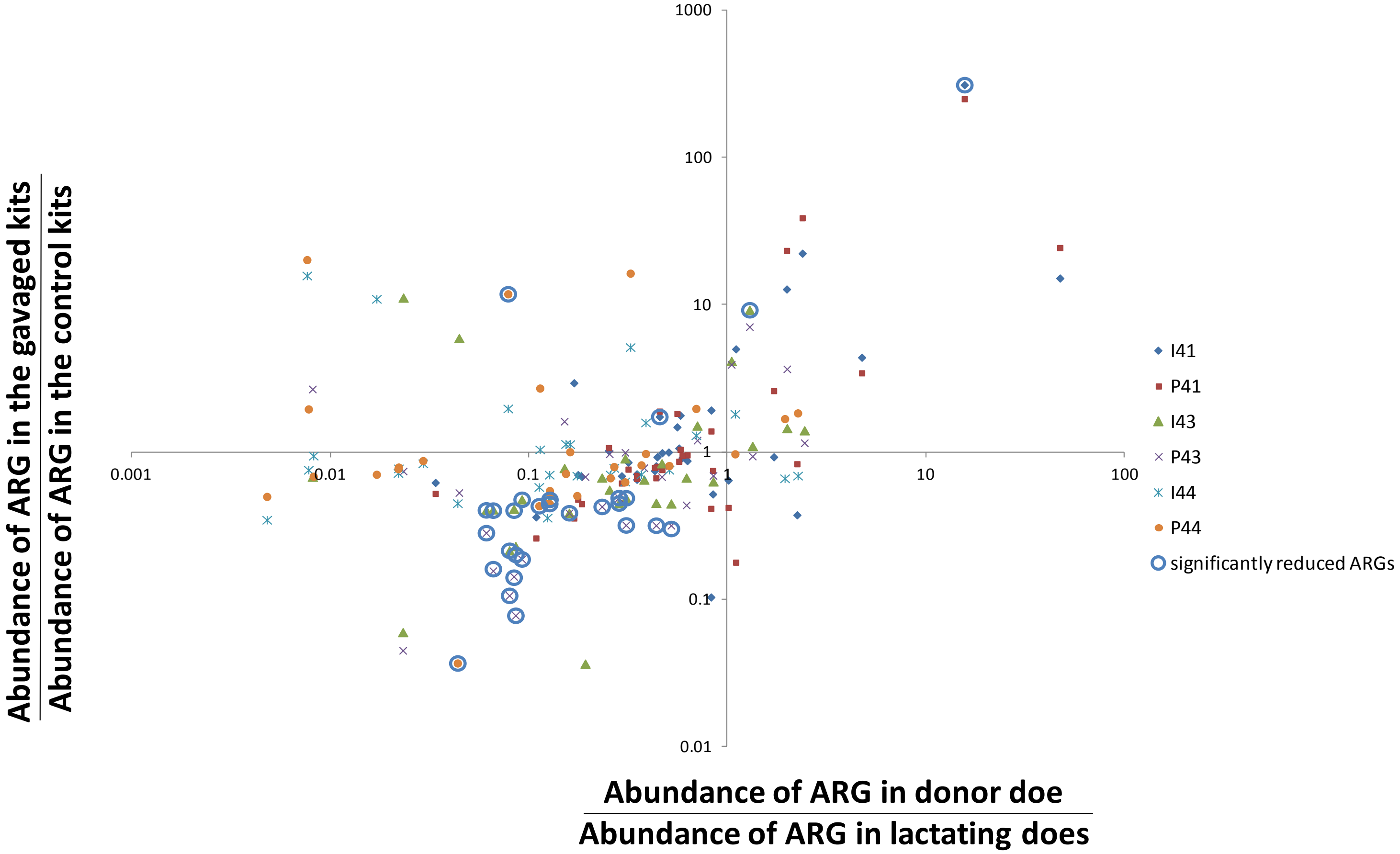

Figure S1: Ratio of abundance of the ARGs in the donor doe and the lactating doe compared with the ratio observed in the gavaged kits vs. the control kits. The ARGs that are significantly impacted are circled. The symbols refer to the groups.

This figure illustrates that predicting the outcome of the competitive exclusion based on the ARGs is challenging. Indeed, the prediction expects all the points to be in the bottom-left or top-right quadrant. Yet, we do find ARGs that are in the top-left quadrant, meaning that they are depleted in the donor doe (compared to the lactating dam), and still more abundant in the gavaged kits (in comparison to the control kits).

5 Additional details about the neutral model

5.1 Rationale of the neutral model

The rationale of the neutral model is that a metacommunity (microbes surrounding the kits in our case) feed a local community (microbes in the gut of the kits). In other words, there is an immigration rate from the metacommunity to the local community. The neutral model assumes that species are 'neutral' in their ecological fitness (i.e., each species has the same growth, death and dispersion rates), and the assembly is the resulting stochastic phenomenon.

The metacommunity can either be objectively measured (such as the microbiota of the lactating dam and the microbiota of the donor doe), or it can be derived from the observation across all the samples. The basic idea is that an operational taxonomic unit (OTU) that is rare in the metacommunity will be observed in very few local communities, whereas an OTU that is abundant in the metacommunity will be observed in every local community.

In our case, we first show that the link between the microbiota of the dams and the microbiota of the kits is tenuous since most of the OTUs observed in every kit are not observed in the dams (with an identical sequencing depth, Fig. S4). We then proceed to fit the neutral model by estimating the metacommunity from the observations across all the kits. The interested reader is invited to read the publication describing the algorithm fitting the neutral model : A.R. Burns, W.Z. Stephens, K. Stagaman, S. Wong, J.F. Rawls, K. Guillemin, and B.J. Bohannan, Contribution of neutral processes to the assembly of gut microbial communities in the zebrafish over host development. The ISME journal 10 (2016) 655-64.

5.2 The abundance of the OTU in a kit is not proportional to its abundance in the lactating dam

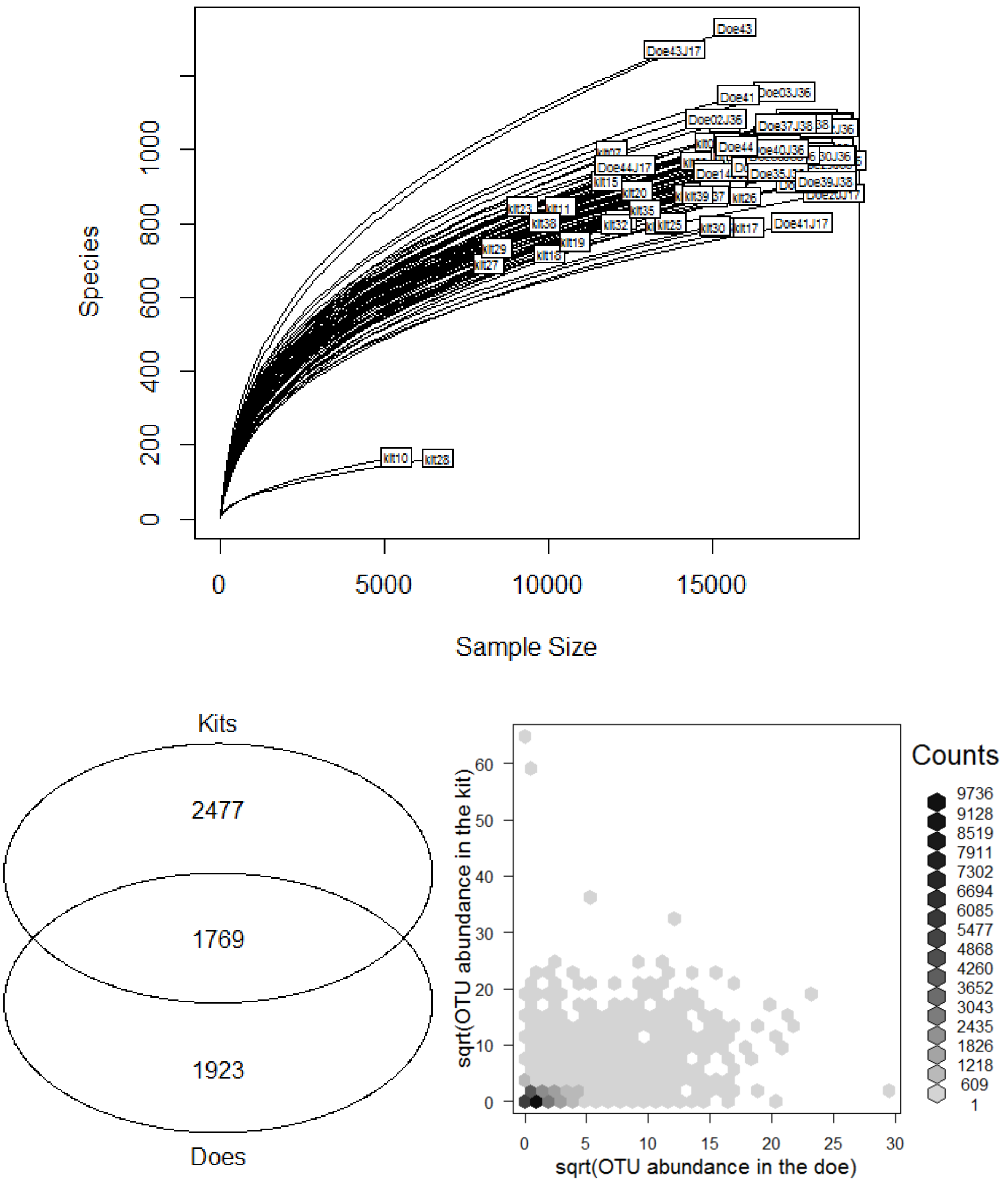

Figure S4: Rarefaction curves and abundance of each Operational Taxonomic Unit (OTU) in the does and the corresponding kit. The rarefaction curves (performed with the 'rarecurve' function of the vegan package (vegan_2.5-4) illustrate how much diversity was captured (TOP PANEL). Relatively few OTUs are common between the 37 dams and the 37 kits (29%) and 58% of the OTUs detected in the kits are absent from the does (including the three donor does) (BOTTOM PANEL). The bottom panel also shows that the abundance of the OTUs in the kits is not simply a reflection of the abundance in the doe (which would be expressed as a straight line).

5.3 Parameter of the neutral model (Table S4)

Table S4: Parameters of the neutral model fitted according to Burns et al. (2016)

group_lab	P44	I44	P43	I43	P41	I41	ControlNF	Control
m	0.41	0.33	0.61	0.39	0.46	0.36	0.59	0.35
m.ci	0.06	0.05	0.09	0.05	0.07	0.06	0.11	0.05
m.mle	0.41	0.33	0.61	0.39	0.46	0.36	0.59	0.35
maxLL	-65.17	-50.46	-28.74	-66.29	-23.24	29.69	67.09	0.46
binoLL	-166.90	-177.27	-156.27	-176.29	-131.71	-87.14	-51.52	-129.12
poisLL	-166.92	-177.29	-156.28	-176.31	-131.72	-87.15	-51.53	-129.13
Rsqr	0.40	0.34	0.33	0.37	0.30	0.19	-0.02	0.27
Rsqr.bino	0.15	0.05	0.20	0.04	0.10	-0.06	-0.10	-0.04
Rsqr.pois	0.15	0.05	0.20	0.04	0.10	-0.06	-0.10	-0.04
RMSE	0.22	0.23	0.23	0.22	0.23	0.25	0.27	0.24
RMSE.bino	0.27	0.27	0.26	0.28	0.27	0.29	0.28	0.29
RMSE.pois	0.27	0.27	0.26	0.28	0.27	0.29	0.28	0.29
AIC	-126.34	-96.92	-53.47	-128.58	-42.48	63.38	138.17	4.93
BIC	-116.99	-87.40	-44.04	-119.07	-33.27	72.55	147.02	14.41
AIC.bino	-329.80	-350.54	-308.54	-348.58	-259.42	-170.28	-99.05	-254.23
BIC.bino	-320.44	-341.02	-299.11	-339.07	-250.21	-161.12	-90.20	-244.75
AIC.pois	-329.84	-350.58	-308.56	-348.62	-259.45	-170.31	-99.05	-254.26
BIC.pois	-320.48	-341.06	-299.13	-339.11	-250.24	-161.14	-90.20	-244.78
N	5297	5313	5318	5312	5314	5311	5312	5311
Samples	5	5	4	5	4	4	3	5
Richness	793	864	826	859	740	724	618	847
Detect	0.000188786	0.000188218	0.000188041	0.000188253	0.000188182	0.000188289	0.000188253	0.000188289
metacomm_lab	metacomm44	metacomm44	metacomm43	metacomm43	metacomm41	metacomm41	metacommNF	metacommControl
Pellet , inoculation or control	P	I	P	I	P	I	C	C

5.4 Strong immigration correlates with strong differences in kits (Fig. S6)

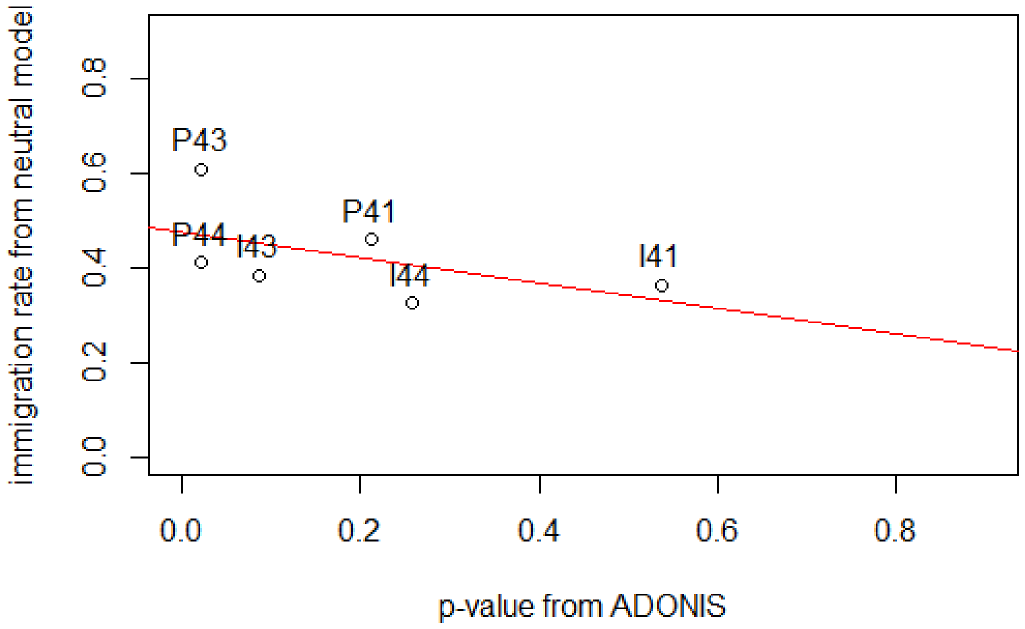

Figure S6: Correlation between the p-value of the ADONIS test of each group vs. the control kits and the immigration rate estimated by the neutral model from the species abundance distribution across the kits: the higher the immigration from the metacommunity is, the more likely it will be different from the control kits (i.e., low p-values, R^2=0.4). The model was fitted to the species abundance distribution from the kits to the estimated metacommunity. The ControlNF group was not taken into account because the neutral model could not estimate an immigration rate for that group (R^2=0). The exact values are given in the table above.

6 Additional details about the networks

6.1 Rationale of separate analysis of kits and dams for the analysis

We used MINE here to determine both linear and non-linear relationships. Since the MINE algorithm detects a non-random distribution of two variables, we analyzed the data of the dams and the kits separately to avoid spurious relationships due to different abundances in does and kits (both tables are available as supplementary data 1 and 2 respectively). In other words, we aim to look at OTU-ARG correlations *within* kits and *within* does. This strategy allows us to detect co-occurrence and exclusion relationships despite the marked difference between does and kits. Indeed using this strategy, we would not falsely identify a relationship between an OTU and an ARG that are both always abundant in does and rare in kits.

6.2 Determination of the significance values for the MINE relationships

To determine the significance of the associations detected by MINE, we corrected for the false discovery rate by running the algorithm on the same matrix randomized per OTU or ARG. In other words, the order of each OTU was permuted and the algorithm was run again. Since all the MINE values obtained in this manner are random by definition, it allows to control the false discovery rate with a realtistic ARG/OTU abundance distribution.

6.3 List of relationships in kits (Table S5)

Table S5: List of relationships that are significant in kits using the MINE methodology (see Materials and Methods section).

	X.var	Y.var	MIC.. strength.	MIC.p.2.. nonlinearity.	MAS.. non.monotonicity.	MEV.. functionality.	MCN.. complexity	Linear. regression.
1	Macrolide_ermB_3	Macrolide_ermB_2	0.99947	0.04760218	0.02039999	0.94541	3	0.97563714
2	Macrolide_ermG.1	Macrolide_ermG	0.99947	0.01969957	0.09755999	0.92501	3	0.98983353
3	Tetracycline_tet40_2	Tetracycline_tet40_1	0.99947	0.10272801	0	0.99947	2	0.9469646
4	OTU4732	OTU3	0.99947	0.04179776	0.13022	0.91176	3	0.9786073
5	Macrolide_ermB_2	Macrolide_ermB	0.92501	0.04309535	0.04559004	0.86826	3	0.9391031
6	Tetracycline_tet40_2	Aminoglycoside_aphA3	0.85361	0.20633727	0.25173	0.7047	3	0.8045326
7	Aminoglycoside_aph2Ib	Aminoglycoside_aac6Im	0.84185	-0.11362964	0.05497998	0.80105	3	0.9774864
8	OTU4268	OTU1	0.84185	-0.11446154	0.04145998	0.64368	3	0.97791183
9	OTU29	beta lactam_CblA1	0.84185	0.09465039	0.08340997	0.74375	3	0.86440706
10	Macrolide_ermB_3	Macrolide_ermB	0.82267	-0.06743348	0.05111998	0.82267	2.5849626	0.94345295
11	Aminoglycoside_strB	Sulfamide_sul2	0.80039	-0.13495445	0.12405002	0.50093	3	0.9671321
12	OTU17	OTU534	0.80039	0.30222157	0.09755999	0.69156	3	0.7058105
13	Aminoglycoside_MGaph	Aminoglycoside_aadE	0.76441	0.6724735	0.38700002	0.56704	3	0.30321038
14	Aminoglycoside_aph3Ib	Aminoglycoside_strB	0.75018	-0.24053216	0.12135005	0.69612	3	0.99534523
15	OTU20	Tetracycline_tet32	0.75018	0.5911374	0.01747	0.66299	3	-0.3988014
16	OTU19	Aminoglycoside_aadE	0.75018	0.7215552	0.14047003	0.6484	3	-0.1691888
17	OTU7340	OTU6	0.74006	0.22187817	0.07433999	0.62816	3	0.71984845
18	OTU70	OTU20	0.72313	0.6358126	0.17759997	0.63103	3	0.29549518
19	OTU1540	OTU9	0.71036	0.2617465	0.12286001	0.68896	3	0.66978616
20	Aminoglycoside_aphA3	Aminoglycoside_aadE	0.70395	0.10274386	0.20187	0.66174	3	0.7753748

21	OTU49	Aminoglycoside_aadE	0.68167	0.66585064	0.24306998	0.57192	3	0.12577507
22	OTU1128	OTU688	0.68167	0.00472385	0.20312	0.64962	3	0.8227674
23	Aminoglycoside_aac6Im	Macrolide_ermB	0.67014	0.53129256	0.24153003	0.43282	3	0.37262243
24	OTU44	OTU40	0.66709	0.65575975	0.36578	0.62297	3	0.10644351
25	OTU22	OTU884	0.66686	0.6668282	0.26059	0.52334	3	0.00563449
26	Tetracycline_tet40_1	Aminoglycoside_aphA3	0.66365	0.06541359	0.14493999	0.59791	3	0.7734574

6.4 Pairwise representation of the relationships detected in the kits (Fig. S7)

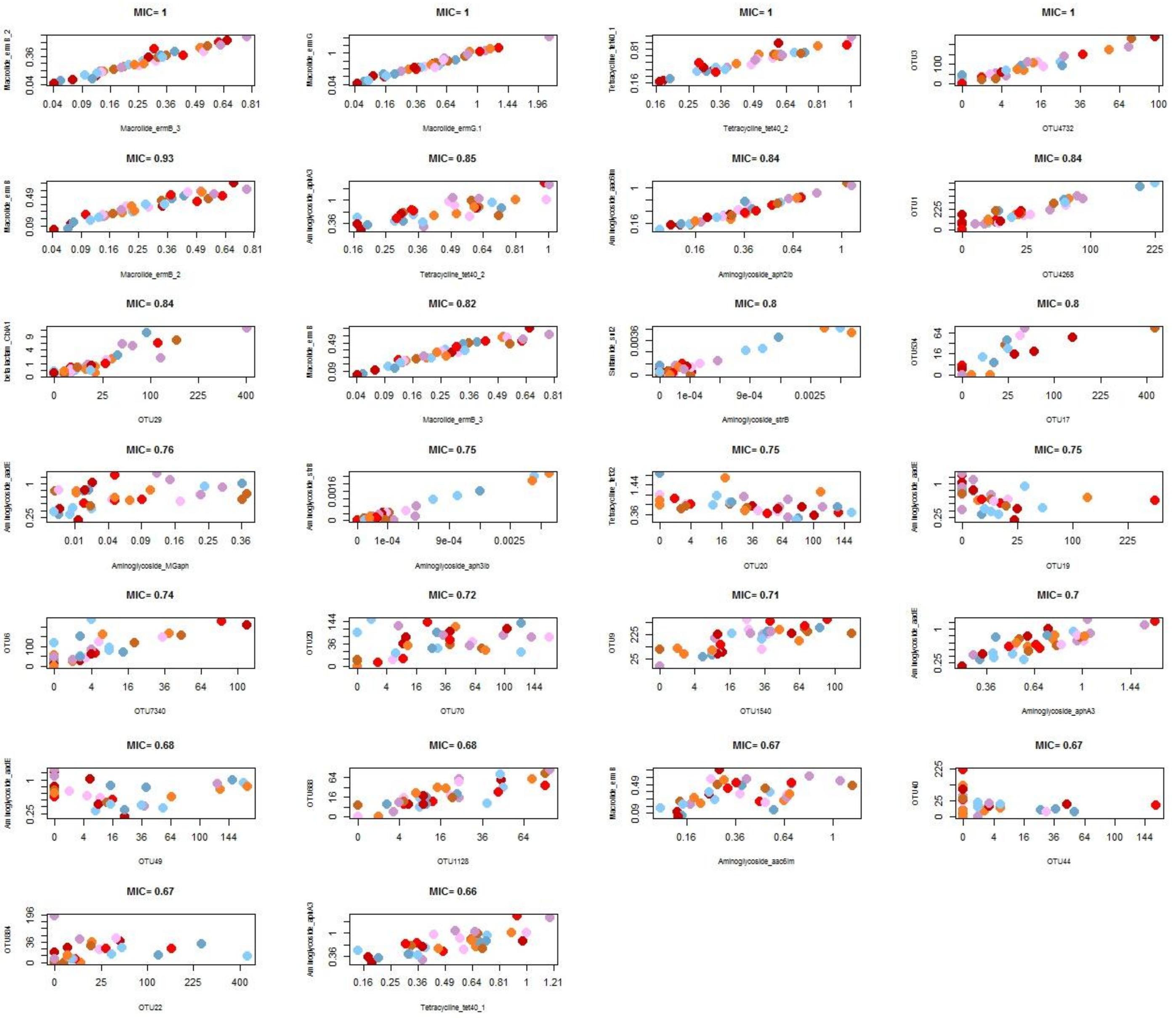

Figure S7: Detail of the pairwise associations found to be significant in the kits. The color indicates the group: purple= control, plum = NF, blue = I41/P41, orange= I43/P43, red = I44/P44

6.5 List of relationship in dams (Table S6)

Table S6: List of relationships that are significant in dams

	X.var	Y.var	MIC.. strength	MIC.p.2.. nonlinearity	MAS.. non.monotonicity	MEV.. functionality	MCN.. complexity	Linear. regression
1	Aminoglycoside_aph2Ib	Aminoglycoside_aac6Im	1	0.09056121	0.09025002	1	3	0.953645
2	Macrolide_ermG.1	Macrolide_ermG	1	0.02166367	0.09025002	1	3	0.989108
3	Macrolide_ermB_3	Macrolide_ermB_2	0.93112	-0.00601965	0.21494001	0.93112	3	0.968059
4	OTU21	betalactam_CblA1	0.90973	0.06361085	0.12993002	0.90973	2.584963	0.919847
5	Macrolide_ermB_2	Macrolide_ermB	0.82727	-0.07094199	0.07073003	0.82727	2.584963	0.947740
6	OTU6557	OTU1	0.8245	0.11131746	0.14752	0.8245	2.584963	0.844501
7	OTU44	OTU11173	0.81269	-0.11097127	0.02433002	0.81269	3	0.961073
8	OTU756	OTU880	0.7965	0.11256057	0.23908001	0.7965	3	0.827006
9	Aminoglycoside_aph3Ib	Aminoglycoside_strB	0.79338	-0.19818246	0.08667004	0.79338	3	0.995772
10	OTU697	OTU800	0.7676	0.23836035	0.13717002	0.7676	3	0.727488
11	Macrolide_ermB_3	Macrolide_ermB	0.76179	-0.14783567	0.08224005	0.76179	3	0.953743
12	OTU697	OTU717	0.73531	0.19482744	0.13567	0.73531	3	0.735175
13	OTU1286	OTU777	0.72768	0.08452481	0.1509	0.72768	3	0.801969
14	OTU717	OTU661	0.71766	0.2151354	0.27737	0.71766	2.584963	0.708889
15	OTU1051	OTU897	0.69327	0.04817873	0.08160001	0.69327	3	0.803175
16	OTU717	OTU777	0.69133	0.14407909	0.08526	0.69133	3	0.739764
17	OTU12442	OTU5	0.6851	0.14683431	0.02715004	0.6851	3	0.733665
18	OTU2989	OTU755	0.68395	0.01677567	0.15293002	0.68395	3	0.816807
19	OTU299	OTU1059	0.67903	0.1372968	0.04534	0.67903	2.584963	0.736025
20	Aminoglycoside_aphA3	Aminoglycoside_aadE	0.67884	0.16816282	0.18436998	0.67884	3	0.714616
21	OTU2989	OTU767	0.67298	0.25488675	0.15687001	0.67298	3	0.646601
22	OTU1472	OTU58	0.67089	0.65156937	0.21131998	0.67089	3	-0.138998
23	OTU1472	OTU777	0.65563	0.15183908	0.27071998	0.65563	3	0.709782
24	OTU715	OTU661	0.65542	0.09481597	0.00937998	0.65542	2.584963	0.748735
25	Tetracycline_MGtetM	Tetracycline_tetM	0.65517	-0.2931525	0.14433002	0.65517	3	0.973818
26	OTU95	Macrolide_ermB_2	0.65504	0.62679297	0.36771002	0.65504	3	0.168068
27	OTU790	OTU755	0.65429	0.19465101	0.03061998	0.65429	3	0.677966

6.6 ARG clusters and ARG-OTU relationships in the does (Fig. S8)

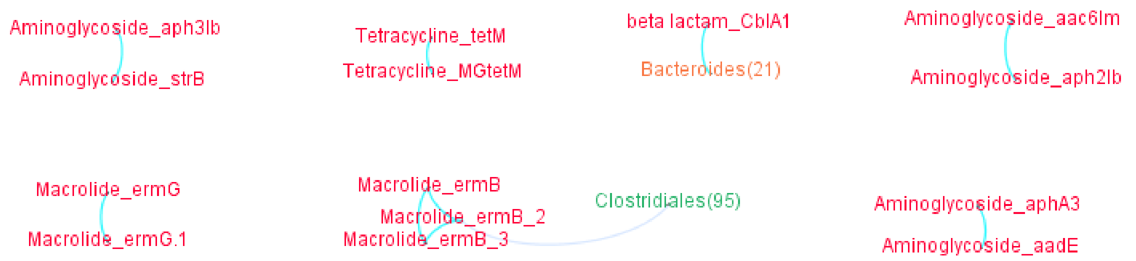

Figure S8: ARG-OTU associations found to be significant in the does. The numbers indicate the OTU numbers. OTU21, affiliated with the *Bacteroides* genus in which this gene was first described. For clarity, only the relationships involving an ARG are plotted.

7 Second trial of competitive exclusion

7.1 Description of the second trial to measure ARG exclusion

At the end of the first trial, the lactating does were inseminated to provide a new litter of kits. In the second experiment, the effects of microbiota I41 and P41 were not tested. For the second experiment, we only determined the proportion of resistant *Enterobacteriaceae*. The gravid does stayed in the same cage as in the first trial but were not given the same treatment. When pooling the data from both trials (i.e., 8-10 kits per group), the I44 group became significant (0.04 instead of 0.07), and the sulfonamide-resistant *enterobacteria* also became significant.

7.2 Raw data of the resistant *Enterobacteria* in both trials

Table S7: Raw data of the resistant *Enterobacteria*

Group	Trial	Cage	Proportion_Tet resistant_Enterobacteria_in_ Does (%)	Proportion_Tet resistant_Enterobacteria_in_ Kits (%)	Proportion_Sul resistant_Enterobacteria_in_ Does (%)	Proportion_Sul resistant_Enterobacteria_in_ Kits (%)
Control	E1	8	65	86	69	86
Control	E1	13	NA	NA	NA	NA
Control	E1	17	88	94	88	100
Control	E1	27	80	100	60	100
Control	E1	35	98	95	100	95
Control	E2	4-global	NA	NA	NA	NA
Control	E2	9-global	NA	NA	NA	NA
Control	E2	14-global	100	55	100	55
Control	E2	20-global	NA	NA	NA	NA
Control	E2	26-global	NA	NA	80	100
Control	E2	30-global	NA	NA	NA	NA
Control	E2	37-global	67	100	67	100
NF	E1	2	100	100	100	100
NF	E1	10	NA	NA	NA	NA
NF	E1	18	75	100	75	100
NF	E1	25	100	83	100	57
NF	E2	1-global	NA	NA	NA	NA
NF	E2	5-global	NA	NA	NA	NA
NF	E2	17-global	93	60	90	45
NF	E2	21-global	NA	NA	NA	NA
NF	E2	27-global	NA	NA	NA	NA
NF	E2	32-global	63	33	63	33
I41	E1	4	NA	NA	NA	NA
I41	E1	11	67	3	67	3
I41	E1	19	70	33	70	30
I41	E1	28	93	100	100	100
I41	E1	37	97	5	100	5
I43	E1	3	53	67	NA	NA
I43	E1	12	53	100	100	100
I43	E1	20	87	5	97	100
I43	E1	29	NA	NA	NA	NA
I43	E1	38	100	2	100	2
I43	E2	7-global	85	33	65	33
I43	E2	13-global	NA	NA	NA	NA
I43	E2	16-global	NA	NA	NA	NA
I43	E2	25-global	NA	NA	NA	NA
I43	E2	35-global	63	2	67	2
I43	E2	39-global	NA	NA	53	2
I44	E1	1	51	2	57	2
I44	E1	5	100	16	100	68
I44	E1	21	100	20	100	20
I44	E1	30	NA	NA	NA	NA
I44	E1	39	NA	NA	88	2
I44	E2	6-global	NA	NA	NA	NA
I44	E2	19-global	93	4	83	52
I44	E2	24-global	55	5	70	75

I44	E2	28-global	57	10	57	5
I44	E2	31-global	NA	NA	NA	NA
I44	E2	36-global	NA	NA	NA	NA
P41	E1	6	100	100	100	100
P41	E1	14	100	30	100	100
P41	E1	22	100	67	100	67
P41	E1	31	NA	NA	NA	NA
P43	E1	7	73	33	78	33
P43	E1	15	NA	NA	NA	NA
P43	E1	23	100	67	97	63
P43	E1	32	100	82	90	89
P43	E2	3-global	NA	NA	NA	NA
P43	E2	10-global	NA	NA	NA	NA
P43	E2	12-global	70	0	90	100
P43	E2	18-global	80	100	85	100
P43	E2	29-global	NA	NA	63	100
P43	E2	40-global	NA	NA	NA	NA
P44	E1	9	100	43	95	46
P44	E1	16	100	2	88	2
P44	E1	26	100	5	100	5
P44	E1	33	100	2	98	2
P44	E1	40	67	2	NA	NA
P44	E2	2-global	NA	NA	NA	NA
P44	E2	11-global	57	4	57	38
P44	E2	15-global	NA	NA	NA	NA
P44	E2	22-global	NA	NA	NA	NA
P44	E2	34-global	NA	NA	NA	NA
P44	E2	38-global	73	37	77	7

7.3 Correspondance of sequencing data

samples	group	animal	nameSequencing
L01	I44	lactatingDoe	CA1501L01J36
L02	ControlNF	lactatingDoe	CA1501L02J36
L03	I43	lactatingDoe	CA1501L03J36
L04	I41	lactatingDoe	CA1501L04J36
L05	I44	lactatingDoe	CA1501L05J36
L06	P41	lactatingDoe	CA1501L06J36
L07	P43	lactatingDoe	CA1501L07J36
L08	Control	lactatingDoe	CA1501L08J36
L09	P44	lactatingDoe	CA1501L09J36
L10	ControlNF	lactatingDoe	CA1501L10J36
L11	I41	lactatingDoe	CA1501L11J36
L12	I43	lactatingDoe	CA1501L12J36
L13	Control	lactatingDoe	CA1501L13J36
L14	P41	lactatingDoe	CA1501L14J36
L15	P43	lactatingDoe	CA1501L15J36
L16	P44	lactatingDoe	CA1501L16J36
L17	Control	lactatingDoe	CA1501L17J36
L18	ControlNF	lactatingDoe	CA1501L18J36
L19	I41	lactatingDoe	CA1501L19J36
L20	I43	lactatingDoe	CA1501L20J36
L21	I44	lactatingDoe	CA1501L21J36
L22	P41	lactatingDoe	CA1501L22J36
L23	P43	lactatingDoe	CA1501L23J36
L25	ControlNF	lactatingDoe	CA1501L25J36
L26	P44	lactatingDoe	CA1501L26J36
L27	Control	lactatingDoe	CA1501L27J36
L28	I41	lactatingDoe	CA1501L28J36
L29	I43	lactatingDoe	CA1501L29J36
L30	I44	lactatingDoe	CA1501L30J36
L31	P41	lactatingDoe	CA1501L31J36
L32	P43	lactatingDoe	CA1501L32J36
L33	P44	lactatingDoe	CA1501L33J36
L35	Control	lactatingDoe	CA1501L35J36
L37	I41	lactatingDoe	CA1501L37J36

L38	I43	lactatingDoe	CA1501L38J36
L39	I44	lactatingDoe	CA1501L39J36
L40	P44	lactatingDoe	CA1501L40J36
L41-J2	D41	donor	CA1501L41J17
L41	D41	donor	CAL41
L42-J2	D42	donor	
L43	D43	donor	CAL43
L43-J2	D43	donor	CA1501L43J17
L44	D44	donor	CAL44
L44-J2	D44	donor	CA1501L44J17
p01-1	I44	kit	CA150101
p02-1	ControlNF	kit	CA150102
p03-1	I43	kit	CA150103
p04-1	I41	kit	CA150104
p05-3	I44	kit	CA150105
p06-3	P41	kit	CA150106
p07-2	P43	kit	CA150107
p08-2	Control	kit	CA150108
p09-2	P44	kit	CA150109
p10-1	ControlNF	kit	CA150110
p11-1	I41	kit	CA150111
p12-1	I43	kit	CA150112
p13-3	Control	kit	CA150113
p14-3	P41	kit	CA150114
p15-1	P43	kit	CA150115
p16-2	P44	kit	CA150116
p17-2	Control	kit	CA150117
p18-1	ControlNF	kit	CA150118
p19-1	I41	kit	CA150119
p20-1	I43	kit	CA150120
p21-3	I44	kit	CA150121
p22-3	P41	kit	CA150122
p23-1	P43	kit	CA150123
p25-1	ControlNF	kit	CA150125
p26-3	P44	kit	CA150126
p27-3	Control	kit	CA150127
p28-1	I41	kit	CA150128
p29-2	I43	kit	CA150129
p30-2	I44	kit	CA150130
p31-1	P41	kit	CA150131
p32-1	P43	kit	CA150132
p33-1	P44	kit	CA150133
p35-3	Control	kit	CA150135
p37-3	I41	kit	CA150136
p38-1	I43	kit	CA150137
p39-2	I44	kit	CA150138
p40-2	P44	kit	CA150139

7.4 Proportion of resistant *Enterobacteria* in both trials (Fig. S2)

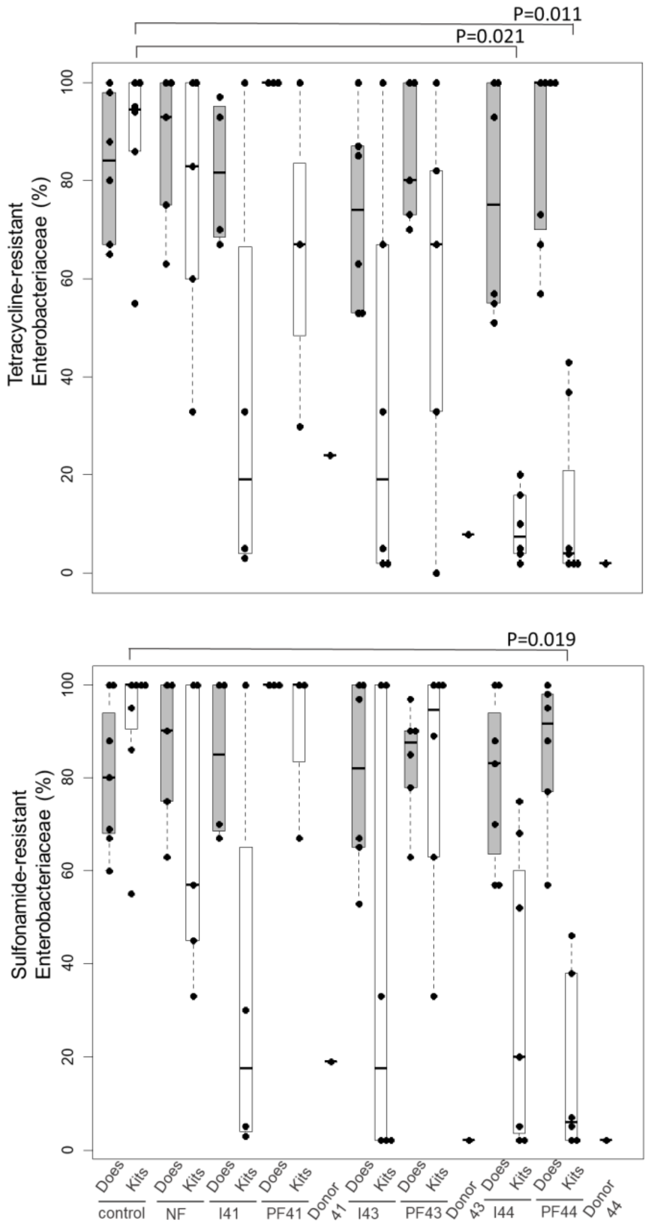

Figure S2: Abundance of *Enterobacteriaceae* isolates resistant to tetracycline and sulfonamides in the eight groups. Controls (C), no-feces controls (NF), Inoculum41 (I41) and fecal pellets of Doe41, Inoculum43 (I43) and fecal pellets of Doe43, Inoculum44 (I44) and fecal pellets of Doe44. The gray bars indicate the proportion of resistant *Enterobacteria* in the lactating dams during the experiment; the white bar shows the resistance in the corresponding kits at weaning. The proportion of resistance in donor does during the experiment is also presented.

This figure shows that adding fecal pellets is slightly more efficient than oral inoculation.
